# NECAB1 and NECAB2 are Prevalent Calcium-Binding Proteins of CB_1_/CCK-Positive GABAergic Interneurons

**DOI:** 10.1093/cercor/bhaa326

**Published:** 2020-11-24

**Authors:** Vivien Miczán, Krisztina Kelemen, Judit R Glavinics, Zsófia I László, Benjámin Barti, Kata Kenesei, Máté Kisfali, István Katona

**Affiliations:** Momentum Laboratory of Molecular Neurobiology, Institute of Experimental Medicine, Budapest 1083, Hungary; Roska Tamás Doctoral School of Sciences and Technology, Faculty of Information Technology and Bionics, Pázmány Péter Catholic University, Budapest 1083, Hungary; Momentum Laboratory of Molecular Neurobiology, Institute of Experimental Medicine, Budapest 1083, Hungary; Department of Physiology, Faculty of Medicine, George Emil Palade University of Medicine, Pharmacy, Science and Technology of Târgu Mureș, Târgu Mureș 540142, Romania; Momentum Laboratory of Molecular Neurobiology, Institute of Experimental Medicine, Budapest 1083, Hungary; Momentum Laboratory of Molecular Neurobiology, Institute of Experimental Medicine, Budapest 1083, Hungary; Szentágothai János Doctoral School of Neuroscience, Semmelweis University, Budapest 1083, Hungary; Momentum Laboratory of Molecular Neurobiology, Institute of Experimental Medicine, Budapest 1083, Hungary; Szentágothai János Doctoral School of Neuroscience, Semmelweis University, Budapest 1083, Hungary; Momentum Laboratory of Molecular Neurobiology, Institute of Experimental Medicine, Budapest 1083, Hungary; Momentum Laboratory of Molecular Neurobiology, Institute of Experimental Medicine, Budapest 1083, Hungary; Momentum Laboratory of Molecular Neurobiology, Institute of Experimental Medicine, Budapest 1083, Hungary; Department of Psychological and Brain Sciences, Indiana University, Bloomington, IN 47405, USA

**Keywords:** basolateral amygdala, Ca^2+^-buffering, cerebral cortex, hippocampus, inhibitory cell

## Abstract

The molecular repertoire of the “Ca^2+^-signaling toolkit” supports the specific kinetic requirements of Ca^2+^-dependent processes in different neuronal types. A well-known example is the unique expression pattern of calcium-binding proteins, such as parvalbumin, calbindin, and calretinin. These cytosolic Ca^2+^-buffers control presynaptic and somatodendritic processes in a cell-type-specific manner and have been used as neurochemical markers of GABAergic interneuron types for decades. Surprisingly, to date no typifying calcium-binding proteins have been found in CB_1_ cannabinoid receptor/cholecystokinin (CB_1_/CCK)-positive interneurons that represent a large population of GABAergic cells in cortical circuits. Because CB_1_/CCK-positive interneurons display disparate presynaptic and somatodendritic Ca^2+^-transients compared with other interneurons, we tested the hypothesis that they express alternative calcium-binding proteins. By in silico data mining in mouse single-cell RNA-seq databases, we identified high expression of *Necab1* and *Necab2* genes encoding N-terminal EF-hand calcium-binding proteins 1 and 2, respectively, in CB_1_/CCK-positive interneurons. Fluorescent in situ hybridization and immunostaining revealed cell-type-specific distribution of NECAB1 and NECAB2 throughout the isocortex, hippocampal formation, and basolateral amygdala complex. Combination of patch-clamp electrophysiology, confocal, and STORM super-resolution microscopy uncovered subcellular nanoscale differences indicating functional division of labor between the two calcium-binding proteins. These findings highlight NECAB1 and NECAB2 as predominant calcium-binding proteins in CB_1_/CCK-positive interneurons.

## Introduction

Ionized calcium (Ca^2+^) is the most versatile intracellular messenger. To subserve pleiotropic physiological functions, Ca^2+^-signaling dynamics must be tightly controlled in a spatially and temporally restricted manner (Berridge et al. 2000). A myriad of proteins, the so-called “Ca^2+^-signaling toolkit,” were evolved to mediate and regulate Ca^2+^-entry, cytosolic free Ca^2+^-levels, and Ca^2+^-extrusion/uptake. A particularly large protein family, the EF-hand Ca^2+^-binding protein superfamily consists of several hundred proteins that play various physiological roles as Ca^2+^-sensors and/or Ca^2+^-buffers (Kawasaki and Kretsinger 2017; Schwaller 2020). Notably, each of the 249 EF-hand Ca^2+^-binding proteins encoded in the mouse genome show characteristic distribution patterns in the brain (Girard et al. 2015). The molecular and anatomical diversity together with the highly different Ca^2+^-binding kinetics of these proteins indicate that cell-type-specific regulation of the spatio-temporal properties of Ca^2+^-signaling is essential for specific computational functions in brain circuits. However, the cellular complexity in the brain represents a major challenge and hence our knowledge about how the specific molecular components of the “Ca^2+^-signaling toolkit” determine distinct physiological functions has remained rather limited in most cell types.

The cell-type-specific expression of cytosolic Ca^2+^-buffers belonging to the EF-hand superfamily, such as parvalbumin, calbindin, and calretinin has been widely demonstrated in cortical circuits. Visualization of the calcium-binding proteins by immunostaining paved the way for the characterization of the synaptic afferents and postsynaptic targets of major GABAergic interneuron types (Kosaka et al. 1987; Hendry et al. 1989; Gulyás and Freund 1996; Gulyás et al. 1996, 1999). Moreover, these neurochemical markers also turned out to be instrumental later to study the pathological reorganization of the synaptic inputs and outputs of GABAergic cell types in brain disorders, such as epilepsy and schizophrenia (Wittner et al. 2001, 2002; Tóth et al. 2010; Chung et al. 2016). In addition, electrophysiological and calcium imaging experiments demonstrated that these calcium-binding proteins play important physiological roles in establishing interneuron-specific temporal dynamics and spatial extent of axonal and somatodendritic Ca^2+^-signaling. For example, presynaptic parvalbumin concentration calibrates nanodomain coupling between calcium influx and neurotransmitter release (Eggermann and Jonas 2012). Moreover, the Ca^2+^-buffering capacity of parvalbumin sharpens the time course of local Ca^2+^-signals around postsynaptic microdomains during trains of synaptic inputs, and also extends the temporal window for synaptic integration by prolonging the decay of somatodendritic Ca^2+^-transients in cortical and hippocampal fast-spiking basket cells (Goldberg et al. 2003; Aponte et al. 2008). These subcellular compartment-specific functions of parvalbumin support the temporal precision of rhythmic perisomatic inhibition mediated by the fast-spiking basket cells targeting the somatic region of principal cells (Freund and Katona 2007) that is essential for the generation of synchronized network activity in cortical circuits (Bartos et al. 2007; Cardin et al. 2009; Sohal et al. 2009; Gulyás et al. 2010; Kvitsiani et al. 2013).

In contrast to the well-described physiological functions of parvalbumin-mediated Ca^2+^-buffering and parvalbumin-containing interneurons, how the kinetic properties of Ca^2+^-signaling determine the activity and functional importance of another major cortical interneuron type, the so-called CB_1_/CCK-positive interneuron has remained largely elusive (Freund and Katona 2007). Interestingly, these interneurons outnumber parvalbumin-positive cells in several cortical areas (Whissell et al. 2015; Nguyen et al. 2020) and form ~40% of cortical GABAergic axon terminals including also those that target the perisomatic domain of pyramidal cells (Szabó, Papp, et al. 2014b; Takács et al. 2015). Moreover, regular-spiking CB_1_/CCK-positive interneurons exhibit distinct presynaptic Ca^2+^-dynamics and dendritic Ca^2+^-buffering capacity compared with fast-spiking parvalbumin-containing interneurons (Rózsa et al. 2004; Hefft and Jonas 2005; Aponte et al. 2008; Kisfali et al. 2013; Lőrincz et al. 2016). Thus, it is conceivable to hypothesize that these inhibitory cells are equipped with unique molecular components of the “Ca^2+^-signaling toolkit.” However, a representative EF-hand calcium-binding protein that may shape cell-type-specific Ca^2+^-signaling dynamics in CB_1_/CCK-positive interneurons has not been identified yet.

To address this issue, we carried out a targeted in silico search among all Gene Ontology (GO)-predicted potential calcium-binding proteins in the mouse genome. Our data mining in CB_1_/CCK-positive interneuron expression datasets identified *Necab1* and *Necab2* genes encoding N-terminal EF-hand calcium-binding proteins 1 and 2, respectively, as candidates. Subsequent RNAscope-based fluorescent in situ hybridization (ISH) and immunostaining determined NECAB1 and NECAB2 as ubiquitous calcium-binding proteins in all CB_1_/CCK-positive interneurons throughout the isocortex, the hippocampal formation and the basolateral amygdala (BLA) complex. Furthermore, stochastic optical reconstruction microscopy (STORM) super-resolution imaging in biocytin-filled cells showed a striking presynaptic accumulation of NECAB2 that contrasted the preferential dendritic distribution of NECAB1. Our findings describe NECAB1 and NECAB2 as two major EF-hand calcium-binding proteins in CB_1_/CCK-positive interneurons providing insights into the molecular components that contribute to subcellular compartment- and interneuron-type-specific Ca^2+^-signaling mechanisms.

## Materials and Methods

### In Silico Analysis

A public mRNA expression database obtained from single-cell RNA-sequencing (RNA-seq) performed at the Karolinska Institute (Zeisel et al. 2015) was used to search for the calcium-binding proteins that are highly expressed by the CB_1_/CCK-positive interneurons in the hippocampal CA1 region and in the somatosensory cortex. Interneurons were first selected from the database using their previous sample annotation as GABAergic cells (Zeisel et al. 2015). Because a few copies of mRNA may occasionally appear for each gene when measured by RNA-seq approaches, only those cells were considered in the next filtering step that had *Cnr1* mRNA (encoding the CB_1_ cannabinoid receptor protein) levels exceeding the 10% threshold of the highest *Cnr1*-expressing cell per brain region to avoid false positive cells in the sample. A Grubb’s test was also performed (iteration = 1, α = 0.05) for outlier detection. In the filtered cell population representing the CB_1_/CCK-positive interneurons, the expression levels of all genes (*n* = 660) that may have potential calcium-binding functions according to their GO term (Ashburner et al. 2000; Carbon et al. 2009, 2017) have been measured (database accessed: 23 February 2017). To avoid potential biases due to differences in cell-specific mRNA harvesting efficiency, the mRNA copy number value for individual genes in a given cell was normalized to the mean of the total mRNA copy number per cell in the dataset. Spearman’s rank correlation coefficients of the mRNA levels of “Necab” genes and other selected genes were calculated on the total pool of hippocampal GABAergic interneurons (*n* = 126) found in the database. The following annotations in Zeisel et al. (2015) were used for comparison of *Cnr1* mRNA levels (IN: “Int5” and “Int6” subtypes; PC: “CA1Pyr1”; “CA1Pyr2” subtypes from the hippocampus and “S1Pyr DL/L23/L4/L5/L5a/L6/L6b” subtypes from the somatosensory cortex).

### Preparation of Tissue Sections

All animal experiments were approved by the Hungarian Committee of the Scientific Ethics of Animal Research (license number: PE/EA/354-5/2018) and were performed according to the Hungarian Act of Animal Care and Experimentation (1998, XXVIII, Section 243/1998, renewed in 40/2013), which are in accordance with the European Communities Council Directive of 24 November 1986 (86/609/EEC; Section 243/1998). Mice were kept under approved laboratory conditions and all efforts were made to minimize pain and to reduce the number of animals used.

### Perfusion

Male C57BL/6 mice (postnatal day 50–62, *n* = 6) were used for fixation via transcardial perfusion. After brief isoflurane (Aesica Queenborough Ltd, B506) anesthesia, Avertin (2,2,2, −tribromoethanol, Sigma, T48402; 2-methyl-2-butanol, Sigma, 152 463 in water; 0.4–0.8 mL; 1.25%) was injected intraperitoneally to induce deep anesthesia. Mice were perfused transcardially with 0.9% saline (Molar Chemicals, 07220-101-190) for 2 min, following with 4% paraformaldehyde (PFA, TAAB, P001) dissolved in 0.1 M phosphate buffer (PB, pH = 7.4 containing Na_2_HPO_4_, Sigma, 71 500 and NaH_2_PO_4_, Sigma, 71 500) for 20 min by using approximately 5 mL/min pump speed. After perfusion, the brains were removed from the skull and postfixed for 2 h or overnight (for GAD67 and PV-immunostaining) in 4% PFA, then 50 μm coronal sections were cut in PB using a Leica VT-1200S (Nussloch, Germany) vibratome.

### Biocytin-Filling of Identified Interneurons

In order to visualize single CB_1_/CCK-positive interneurons, adult male C57BL/6 mice (postnatal day 28–35, *n* = 12) were decapitated during isoflurane anesthesia, then brains were quickly removed from the skull and 300 μm coronal sections were cut in ice-cold sucrose containing artificial cerebrospinal fluid (ACSF): 75 mM NaCl, 75 mM sucrose (Sigma, S7903), 2.5 mM KCl (Sigma, P3911), 25 mM glucose (Sigma, G8270), 1.25 mM NaH_2_PO_4_, 4 mM MgCl_2_ (Sigma, M2670), 0.5 mM CaCl_2_ (Sigma, 223 506), and 24 mM NaHCO_3_ (Sigma, S5761) using a Leica VT-1200S vibratome. Slices were pre-incubated at 34 °C in sucrose-containing ACSF for 1 h before placed to a submerged recording chamber containing standard ACSF (2.5 mM KCl, 10 mM glucose, 126 mM NaCl, 1.25 mM NaH_2_PO_4_, 2 mM MgCl_2_, 2 mM CaCl_2_, and 26 mM NaHCO_3_). During both preincubation and recording, ACSF solutions were oxygenated with 95% O_2_ and 5% CO_2_. Electrophysiological recordings were performed at 33 °C, and slices were visualized by a Nikon Eclipse FN1 microscope with infrared differential interference contrast (DIC) optics. Whole-cell patch-clamp recordings were obtained using borosilicate glass pipettes (3–4 MΩ) filled with an intracellular solution containing 126 mM K-gluconate (Sigma, G4500), 4 mM KCl, 10 mM HEPES (Sigma, H4034), 4 mM MgATP (Sigma, A9187), 0.3 mM Na_2_GTP (Sigma, 51 120), 10 mM PO-creatine (Sigma, P7936), and 0.2% biocytin (Sigma, B4261); pH 7.2, 270–290 mOsm. Interneurons with a large cell body and 2–3 major dendrites were selected for patch-clamp recording in the stratum radiatum of the CA1 subfield using the DIC image. All selected cells displayed an accommodating, regular firing pattern and multipolar morphology implicating a CB_1_/CCK-positive interneuron phenotype (Lee et al. 2015). Recordings were performed using MultiClamp700B amplifiers (Molecular Devices, San Jose, CA, USA). Signals were filtered at 3 kHz using a Bessel filter and digitized at 10 kHz with a Digidata analog-digital interface (Molecular Devices). Recorded traces were analyzed using Clampfit 10 software (Molecular Devices). After ~30 min of stable recording, slices were fixed immediately in 4% PFA in PB at 4 °C for 24 h.

### Morphological Analysis of Biocytin-Filled Interneurons

After thorough washing in PB and permeabilization of cell membranes with 30 min incubation in a 0.5% Triton X-100 (Sigma, T98787-50ML) PB solution, single-cell biocytin staining was developed with Alexa-488-conjugated streptavidin (50 μL/mL, Jackson, 016-540-08) treatment of the 300 μm-thick electrophysiological slices. After several washing steps in PB, the slices were mounted onto glass slides in Vectashield (Vector Laboratories, H-1000-10), coverslipped and sealed with nail polish.

To define the specific interneuron type based on morphological criteria, confocal microscopy was used. Confocal *z*-stacks (2048 × 2048 × 150 voxels, voxel size: 0.03 × 0.03 × 1 μm) containing the filled interneuron in the center were acquired with a confocal microscope (NIKON-C2) by using 488 nm illumination. Subsequently, maximal intensity projections (MIPs) were generated to provide a more complete morphological image of the axonal arbor of the respective cell. To distinguish perisomatically targeting and dendritically targeting CB_1_/CCK-positive interneurons, the Bouton distribution index (BDI) was calculated as described earlier (Dudok et al. 2015). Briefly, cells with a BDI > 1 value were categorized as perisomatically targeting cells (the axon arbor was mainly restricted to the stratum pyramidale of the hippocampal CA1 region), interneurons with a BDI < 0.5 were identified as dendritically targeting interneurons (the axon arbor was mainly distributed in both the strata oriens and radiatum). A few morphologically ambiguous cells with 0.5 < BDI < 1 values were omitted from further analysis. After cell type identification, the 300 μm-thick electrophysiological slices were removed from the glass slides, washed extensively in PB and embedded in 2% agarose (Sigma, A9539). Resectioning to 10 or 20 μm thickness to aid antibody penetration in subsequent immunostaining was done in PB with a Leica VT-1200S vibratome.

### Multiplex Fluorescent In Situ Hybridization

RNAscope multicolor ISH was utilized, because this approach enables simultaneous signal amplification and background suppression, and it is capable for highly sensitive single-molecule visualization of mRNA molecules while preserving tissue morphology (Wang et al. 2012). Adult C57BL/6 mice (postnatal day 60–70, *n* = 3) were decapitated under Avertin anesthesia. Mouse brains were rapidly dissected over ice and frozen in isopentane (Sigma, 277 258) on dry ice for 20 s. After embedding in optimal cutting temperature (OCT, Tissue-Tek, 4583) gel, a cryostat (Thermo Scientific, Microm GmbH, HM550) was used to cut 16 μm thick coronal sections at −20 °C. Sections were fixed onto Superfrost Ultra Plus slides (ThermoFisher, J488AMNZ) with 10% PFA in PB and a series of dehydration steps was performed by using 50%, 70%, and kept in 100% ethanol overnight (Molar Chemicals, 02910-101-340). Next day, the sections were dried, and a hydrophobic barrier was drawn around the slices with a hydrophobic pen (ImmEdger Hydrophobic Barrier, Vector Laboratories) for isolation. The sections were pretreated according to the RNAscope kit protocol to permeabilize the cells and unmask the target RNA. Then, the samples were incubated in a HybEZ hybridization oven at 40 °C with 150 μL of specific RNAscope probes for 2 h (Table 1). Washing was performed by immersing the samples in 1× wash buffer. A volume of 150 μL of the Detection Reagent (AMP1, AMP2, AMP3, AMP4) was used to intensify the signal. To mark the cell nuclei, a 30 min-long treatment with 4′,6-diamidino-2-phenylindole solution (DAPI; 1:1000; Calbiochem, 508 741) dissolved in 0.05 M Tris-buffered Saline (TBS, pH = 7.4, Trizma hydrochloride, Sigma, T3253, Trizma base, Sigma, T1503 and NaCl) was performed. Samples were covered in Hardset Vectashield antifade medium (Vector Laboratories, H-1400-10) and sealed with nail polish.

**Table 1:** RNAscope probes used in fluorescent ISH

Target	Cat. No.	Fluorescent amplification
*Necab1*	428 541	Alexa 488
*Necab2*	467 381-C3	Atto 647
*Cnr1*	420 721-C2	Atto 550

Note: Referred in Materials and Methods/Multiplex Fluorescent In Situ Hybridization section.

In some experiments, RNAscope fluorescent ISH was combined with fluorescent immunostaining. After finishing the RNAscope assay, the sections were incubated in 4% PFA for 10 min, then washed in 0.05 M TBS supplemented with 0.1% Triton X-100. Blocking the nonspecific binding sites was done with 5% Normal Donkey Serum (NDS, Sigma, D9663) in TBS. Sections were next incubated in the respective primary antibody (Table 2) and DAPI (1:1000 concentration) containing TBS-solution at 4 °C overnight. The two primary antibodies against NECAB1 and NECAB2 have also been validated by antigen adsorption tests (Zhang et al. 2014) and in case of the antibody against NECAB2 in NECAB2-knockout mice (Zhang et al. 2018). On the next day after TBS washing steps, a 1 h-long incubation followed in a TBS-solution containing the respective fluorescent secondary antibody (Table 2). After washing steps, the sections were mounted in Hardset Vectashield antifade medium and sealed with nail polish.

**Table 2:** RNAscope probes and antibodies used for combined ISH and immunostaining

	NECAB1	NECAB2
RNAscope probes	*Necab1-*428 541	*Necab2-*467 381-C3
Primary antibodies	Rabbit anti-NECAB1 1:500 concentration (Atlas Antibodies, HPA023629)	Rabbit anti-NECAB2 1:500 concentration (Atlas Antibodies, HPA013998)
Fluorescent amplification of the RNAscope channel	Alexa 488	Atto 647
Secondary antibody	Donkey anti-Rabbit Alexa 594 concentration 1:400 (Jackson, 711-585-152)	Donkey anti-Rabbit Alexa 488 concentration 1:400 (Jackson, 711-545-152)

Note: Referred in Materials and Methods/Multiplex Fluorescent In Situ Hybridization section.

### Immunostaining

Fluorescent immunolabeling was used to visualize protein distribution in brain section as described (Barna et al. 2016). Briefly, all sections were immunostained in a free-floating manner in 12- or 24-well tissue culture plates (Greiner Bio-One CELLSTAR 12- or 24-well suspension culture plate, 665 102, 662 102) in 500–1000 μL volume on an orbital shaker (Biosan OS-10). Wells were preblocked with 1% Bovine Serum Albumin (BSA, Sigma-Aldrich, A2153) diluted in TBS to prevent the sections from sticking to the walls. After extensive washing in PB and TBS, sections were treated with a solution containing 5% NDS and 0.1–0.3% Triton X-100 in TBS for 45 min–1 h for blocking nonspecific binding sites and for enhancing antibody penetration, respectively.

Sections were then incubated with the primary antibodies (Table 3) in TBS-solution at room temperature or in 0.1 M PB buffer at 4 °C overnight. Next day, the sections were thoroughly washed and incubated with fluorescently-labeled secondary antibodies (Table 3) in TBS-solution for 4 h. After antibody incubation, the sections were washed in TBS and PB. Finally, the sections were either postfixed for 10 min in 4% PFA and mounted and dried on coverslips for subsequent super-resolution imaging or were mounted in Vectashield supplemented with DAPI (Vector Laboratories, H-1200-10), covered by a coverslip and sealed with nail polish for subsequent confocal microscopy. In case of GAD67- and PV-immunostaining, all incubation steps were performed in 0.1 M PB and DAPI (Sigma, 5 087 410 001) was applied together with the secondary antibody. Sections were mounted in Hardset Vectashield antifade medium (Vector Laboratories, H-1400-10).

**Table 3:** Primary and secondary antibodies used in immunostaining

Antibody type	Target	Species	Concentration	Source
Primary antibodies	Anti-CB_1_	Guinea pig	1:2000	Fukudome et al. (2004)
	Anti-NECAB1	Rabbit	1:300	Atlas Antibodies, HPA023629
	Anti-NECAB2	Rabbit	1:500	Atlas Antibodies, HPA013998
	Anti-CCK	Mouse	1:3000	CURE, 39161
	Anti-GAD67	Mouse	1:2000	Merck, MAB5406
	Anti-PV	Mouse	1:5000	Swant, 235
Secondary antibodies	Anti-guinea pig, Alexa 488-conjugated	Donkey	1:400	Jackson, 706-545-148
	Anti-rabbit Alexa 594-conjugated	Donkey	1:400	Jackson, 711-585-152
	Anti-mouse Alexa 488-conjugated	Donkey	1:400	Jackson, 715-545-150
	Anti-guinea pig Alexa-594-conjugated	Donkey	1:400	Jackson, 706-585-148
	Anti-rabbit Alexa 647-conjugated	Donkey	1:400	Jackson, 711-605-152
	Anti-rabbit Alexa 488-conjugated	Donkey	1:400	Jackson, 711-545-152
	Anti-mouse Alexa 594-conjugated	Donkey	1:400	Jackson, 715-585-150
	Anti-mouse Alexa 647-conjugated	Donkey	1:400	Jackson, 715-605-150

Note: Referred in Materials and Methods/Immunostaining section.

### Confocal Image Acquisition and Analysis of Fluorescent In Situ Hybridization

A NIKON-A1R confocal microscope equipped with a 60× oil-immersion objective was used to obtain high-resolution (500 nm/pixel) images of fluorescent ISH in the hippocampus, the somatosensory cortex, and the BLA complex. Freehand cellular regions of interests (ROIs) were selected based on the overlaid *Cnr1* RNAscope and DAPI signals with the NIS Elements AR analysis software. ROI signal intensities were normalized to the background mean confocal signal intensities to obtain relative enrichment values for each interneuron. Cells were then categorized into “strong-CB_1_” and “weak-CB_1_” groups based on this enrichment value and topological information. The “strong-CB_1_” cells were considered as the putative GABAergic interneurons, whereas the “weak-CB_1_” cells were considered as the putative principal cells in all three regions (enrichment value cutoffs based on distribution of enrichment values per brain region: HC-CA1: 2, HC-CA3: 4.5, HC-DG: 3, SS-CTX: 3, BLA: 5) based on prior findings describing telencephalic *Cnr1* mRNA expression levels in the different cell types (Marsicano and Lutz 1999). The *Necab1* and *Necab2* enrichment values were also obtained from the selected individual cells and plotted. Finally, each cell was categorized based on its *Necab1* and *Necab2* content to establish the ratio of “strong-CB_1_”- and “weak-CB_1_”-expressing cells that are also positive for *Necab1* and/or *Necab2*.

### Confocal Image Acquisition and Analysis of Fluorescent Immunostaining

A NIKON A1R microscope was used with 20× and 60× objectives to obtain high-resolution *z*-stacks of fluorescent immunostainings in the hippocampus, the somatosensory cortex, and the BLA complex. Cell bodies immunostained for CB_1_ receptors were selected manually and their NECAB1- or NECAB2-immunopositivity was evaluated. In case of the biocytin-filled hippocampal interneurons, separate anatomical sections were obtained during the reslicing of the electrophysiological slice preparation and used either for CB_1_- or NECAB1- or NECAB2-immunostaining. The presence of presynaptic CB_1_ receptors was verified in the axon terminals in case of all biocytin-filled regular-spiking multipolar interneurons. NECAB1- and NECAB2-immunostaining were also present in all identified interneurons and their levels were evaluated in the somatic, the dendritic, and the axonal compartments.

### Correlated Stochastic Optical Reconstruction Microscopy and Confocal Microscopy

In order to perform STORM super-resolution imaging on biocytin-filled cells, the electrophysiological slices were resectioned into 10 μm-thick sections and used for subsequent immunostaining as described earlier (Barna et al. 2016). The sections were covered with 25 μL of freshly prepared Smart Buffer (Abbelight) imaging medium, then sealed with nail polish. Image acquisition was conducted by a NIKON Ti-E inverted microscope equipped with a NIKON N-STORM system, C2 scanner head and an Andor iXon Ultra 897 EMCCD camera. Images were obtained with a CFI Apo TIRF 100× objective (NA 1.49). Imaging parameters were controlled by a NIKON NIS-Elements AR software equipped with N-STORM module. STORM image acquisition was done by a 300 mW laser (VFL-P-300-647, MPB Communications, Montreal, Canada), activation was triggered with a Melles Griot 56RCS/S2780 diode laser. Dendrites and axon terminals belonging to the biocytin-filled interneurons were identified by live 488 nm illumination, then confocal *z*-stacks (512 × 512 × 15 voxels, 80 × 80 × 150 nm voxel size) were obtained with 488 and 647 nm illumination from randomly selected ROIs of the subcellular compartments. For super-resolution imaging of NECAB1- or NECAB2-immunostaining, the directSTORM approach was used (Heilemann et al. 2008), and the images were captured during 5000 cycles with 30 ms exposition time with a STORM filter cube. Oblique illumination was obtained using a total internal reflexion fluorescence (TIRF) illuminator. The 3D STORM image acquisition was obtained with a cylindrical lens (Huang et al. 2008). Imaging stability was provided by using a perfect focus system (PFS).

Confocal *z*-stacks were deconvolved with the Huygens Professional 4.2.1 software (SVI, Netherlands) using Classic Maximum Likelihood Estimation algorithm and a theoretical point spread function (PSF) with 100 iterations. Image analysis and ROI selection were conducted on the MIP of the central three images of the confocal stack. STORM coordinates were obtained from STORM images with NIS-Elements AR software N-STORM module. The software uses the 3D-DAOSTORM algorithm for peak detection (Holden et al. 2011; Babcock et al. 2012).

Correlated confocal and STORM super-resolution image analysis was carried out by using the VividSTORM software (Barna et al. 2016). First, STORM coordinates were manually aligned with the confocal image of the NECAB1 or NECAB2 channel, then the ROIs were delineated in an unbiased manner with a Morphological Active Contour Without Edges Algorithm (Marquez-Neila et al. 2014). After a ROI filter was applied to the coordinates, STORM densities were calculated based on the number of localization points (NLP) within the ROI and the size of the ROI. STORM NLP was normalized based on immunostaining density per STORM image to correct for STORM imaging variability. A custom script written in R was used to generate the Ripley’s L functions. The Lest function of the spatstat library (Baddeley and Turner 2005) was used with isotropic border correction and maximal radius of 1000. The previously selected ROIs of NECAB1-dendrites/NECAB1-boutons/NECAB2-dendrites/NECAB2-boutons were placed at distances exceeding their maximal radius, then the Lest function was applied on them.

### Statistical Analysis and Figure Preparation

Statistical analysis was performed with the STATISTICA 13.4 software (TIBCO Data Science, Palo Alto, CA). Sample sizes were estimated based on previous experience and are similar to those generally applied in the field. To determine the appropriate statistical method, data were tested for normality using Kolmogorov–Smirnov test, and differences between animals within a subcellular compartment as group was established by using Kruskal–Wallis test. Differences between the groups were determined using Mann–Whitney *U*-test. Data met the necessary criteria for all analysis used. Figure preparation was done with the Photoshop CS5 (Adobe Systems, San Jose, CA) program. Confocal images of multiple samples presented in the same figure were modified identically in every step to maintain original differences.

## Results

### In Silico Single-Cell RNA-seq Analysis Pinpoints Necab1 and Necab2 as Candidate Genes in CB_1_/CCK-Positive Interneurons

The recent advent of various single-cell RNA-sequencing technologies exposed unprecedented details about the transcriptomic profiles of individual GABAergic interneurons in cortical circuits (Zeisel et al. 2015, 2018; Fuzik et al. 2016; Habib et al. 2016; Tasic et al. 2016; Paul et al. 2017; Harris et al. 2018; Hodge et al. 2019). While these studies primarily focused on cataloging the cellular taxonomy of the hippocampus and the cerebral cortex, emerging expression data also provide an opportunity for hypothesis-driven data mining to identify target genes in specific cell types by using publicly available databases. In order to detect candidate calcium-binding proteins that may shape specific Ca^2+^-signaling dynamics and serve as neurochemical markers of CB_1_/CCK-positive interneurons, we first exploited the original datasets provided by the Karolinska Institute that were obtained from cells in the CA1 subfield of the hippocampus and in the somatosensory cortex of mice (Zeisel et al. 2015). We filtered the datasets including only those GABAergic cells that expressed high levels of *Cnr1* mRNA (for details see Materials and Methods), the gene encoding the CB_1_ cannabinoid receptor, a well-established marker of these interneurons. Importantly, *Cnr1* expression and CB_1_ receptor proteins were previously found to be absent in other major interneuron classes such as the parvalbumin-positive cells in cortical microcircuits (Katona et al. 1999; Marsicano and Lutz 1999; Tsou et al. 1999; Bodor et al. 2005).

As a next step, we collected all potential genes that were annotated with the term “calcium-binding” in the GO knowledgebase (Ashburner et al. 2000; Carbon et al. 2009, 2017). This search revealed 660 candidate genes whose expression levels were measured in the high *Cnr1* expression-selected cell pools obtained from the cortical and hippocampal samples. To test the reliability of the in silico approach, we verified that the three members of the calmodulin calcium-binding family (*Calm1*, *Calm2*, *Calm3*), which are ubiquitously expressed in all eukaryotic cells could be readily detected in both single-cell-based mRNA datasets (Supplementary Fig. 1*a,b*).

**Figure 1:**
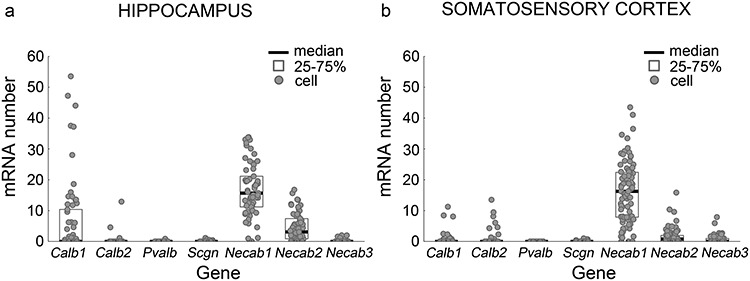
Single-cell RNA-Seq profile of representative calcium-binding proteins reveals high *Necab1* and *Necab2* expression in CB_1_-receptor-expressing GABAergic interneurons. (*a*, *b*) In silico analysis of selected calcium-binding protein encoding mRNA expression profiles was performed on data derived from a publicly available single-cell RNAseq database (Zeisel et al. 2015). The samples were obtained from the mouse hippocampus (*a*) and the somatosensory cortex (*b*). Cells in the database were selected for their GABAergic phenotype and high *Cnr1* expression to focus the analysis on CB_1_/CCK-positive interneurons. The mRNAs of characteristic EF-hand calcium-binding proteins (calbindin—*Calb1*, calretinin—*Calb2*, parvalbumin—*Pvalb* and secretagogin—*Scgn*) are present in low levels in most of these interneurons, and only a few of them exhibit elevated *Calb1* levels. In contrast, the genes encoding the N-terminal EF-hand calcium-binding protein 1 and 2 (*Necab1* and *Necab2*, respectively) are consistently expressed at high levels in all investigated hippocampal and cortical interneurons (*n* = 61 cells in the hippocampus, *n* = 84 cells in the somatosensory cortex).

Subsequently, we analyzed the gene expression levels of the four most common calcium-binding proteins (*Calb1*/calbindin; *Calb*2/calretinin; *Pvalb*/parvalbumin; *Scgn*/secretagogin). These EF-hand calcium-binding proteins display cell-type-specific expression in the brain and have Ca^2+^-buffering functions (Alpár et al. 2012; Schmidt 2012; Schwaller 2014, 2020). In agreement with prior anatomical studies (Marsicano and Lutz 1999; Bodor et al. 2005), *Calb1* expression was noticed in a subpopulation of high *Cnr1* expression-selected cell pools in the hippocampus and in a few cells in the somatosensory cortex (Fig. 1*a,b*). In contrast, *Calb2* expression was rarely observed in the CA1 sample, but was occasionally found in cells derived from the somatosensory cortex (Fig. 1*a,b*), as described earlier (Marsicano and Lutz 1999). Neither *Pvalb* nor *Scgn* expression reached considerable mRNA levels in the high *Cnr1* expression-selected cell pools (Fig. 1*a,b*). These measurements corroborated that the in silico data mining approach is able to replicate previous positive and negative anatomical findings and highlighted that none of the well-studied EF-hand calcium-binding proteins are ubiquitously expressed in cortical CB_1_/CCK-interneurons.

Further manual analysis of the expression datasets revealed two interesting candidate genes, “Necab1” and “Necab2” that were highly and consistently expressed in high “Cnr1” expression-selected cells in both the somatosensory cortex and the hippocampus (Fig. 1*a,b*). In contrast, a third related gene *Necab3* had only very few mRNA copies in these interneurons (Fig. 1*a,b*). *Necab1* and *Necab2* encode N-terminal EF-hand Calcium Binding Protein 1 and 2 (NECAB1 and NECAB2), respectively (Bernier et al. 2001; Sugita et al. 2002). NECAB proteins belong to the EF-hand calcium-binding protein family (Kawasaki and Kretsinger 2017), and comprise an EF-hand domain with a single calcium-binding site at the N-terminal, a central NECAB homology region and a putative antibiotic biosynthesis monooxygenase domain at the C-terminal (Sugita et al. 2002; Wu et al. 2007). Although their specific neuronal function is unknown, both affect cytosolic Ca^2+^-buffering capacity in neuronal preparations (Sugita et al. 2002; Schöndorf et al. 2014). Because both proteins are highly conserved between mouse and human (NECAB1 97%; NECAB2 85% based on UniProt alignment), we wondered if their expression pattern in CB_1_/CCK-positive interneurons is also preserved in humans. Indeed, a search in the single-cell RNA-seq dataset obtained from neuronal nuclei in postmortem human cortical samples by the Allen Institute (Hodge et al. 2019) confirmed that both *NECAB*s are highly expressed in a population of those GABAergic interneurons that originate in the caudal ganglionic eminence (CGE) and have also high expression levels of the human *CNR1* and *CCK* genes. In contrast, *NECAB1* and *NECAB2* expression were very limited or below detection threshold in the other major interneuron class that originates in the medial ganglionic eminence (MGE) and includes the parvalbumin-positive interneuron types (see for details: http://celltypes.brain-map.org/rnaseq/human/cortex). These data together suggest that NECAB1 and NECAB2 are two consensus calcium-binding proteins in CB_1_/CCK-positive interneurons in mammals.

In order to provide an independent line of evidence that elevated *Necab1* and *Necab2* gene expression levels are characterizing features of CB_1_/CCK-positive interneurons, we next calculated the Spearman’s rank correlation coefficient of conventional interneuron marker genes and the *Necab* genes in the total pool of hippocampal GABAergic interneurons (*n* = 126) found in the database (Zeisel et al. 2015). Importantly, *Cnr1* (CB_1_ receptor) and *Cck* (cholecystokinin) gene expression levels reliably predicted *Necab1* and *Necab2* levels in individual interneurons (Supplementary Fig. 2*a*). Moreover, the single-cell mRNA levels of other well-known neurochemical markers that mark major subpopulations of CB_1_/CCK-positive interneurons, such as *Slc17a8* (vesicular glutamate transporter 3, vGluT3), *Sncg* (gamma-synuclein), *Cxcl14* (C-X-C motif chemokine 14) and *Nr2f2* (COUP transcription factor 2, COUP-TFII) also showed significant positive correlation with *Necab1* and *Necab2* levels (Supplementary Fig. 2*a*) (Somogyi et al. 2004; Fuentealba et al. 2010; Lasztóczi et al. 2011; Tasic et al. 2016; Harris et al. 2018; Pelkey et al. 2020). In striking contrast, strong negative correlation was noted between *Necab1*/*Necab2* and *Pvalb* (parvalbumin) or *Sst* (somatostatin) mRNA levels (Supplementary Fig. 2*a*). Because these latter two marker genes define the other major subclasses of MGE-derived GABAergic interneurons (Tasic et al. 2016; Harris et al. 2018), these findings obtained at the single-cell level further underline that *Necab1* and *Necab2* expression distinguishes the CGE-derived CB_1_/CCK-positive interneurons.

Since single-cell mRNA levels of functionally related proteins often covary, we also searched the dataset to detect candidate genes whose physiological function may be associated with NECAB1/NECAB2-mediated Ca^2+^-buffering. This analysis revealed several novel genes with unknown function in CB_1_/CCK-positive interneurons (Supplementary Figs 2*b* and 3). Perhaps the functionally most interesting two genes that exhibited strong correlations with *Necab1* and *Necab2* both encode calcium-sensing proteins that are known to regulate vesicular exocytosis such as *Cadps2* (Calcium Dependent Secretion Activator 2) and *Syt6* (synaptotagmin 6). These observations raise the possibility that NECAB proteins may have presynaptic function and provide further hints that a unique molecular apparatus controls Ca^2+^-dependent neurotransmitter release in CB_1_/CCK-positive interneurons.

### RNAscope Multiplex Fluorescent In Situ Hybridization and Immunostaining Demonstrate that NECAB1 and NECAB2 are Present in All CB_1_/CCK-Positive Interneurons

Although the reproducibility of single-cell RNA-seq approaches has been substantially improved, false positive and false negative hits may both appear in the detection of differentially expressed genes due to inherent technical limitations such as contamination during cell harvesting from tissue preparations or high detection threshold for genes expressed at low levels (Vieth et al. 2019; Wang et al. 2019). For example, *Necab1* was recently found to be a candidate marker of D_1_ dopamine receptor-expressing GABAergic medium spiny projection neurons in the striatum, but later was reported to be enriched in D_2_ dopamine receptor-expressing GABAergic medium spiny projection neurons by using the different single-cell RNA-seq approaches Drop-seq and Nuc-seq, respectively (Saunders et al. 2018; Märtin et al. 2019). In addition, mRNA transcription in a single cell does not always accurately predict protein translation (Sharma et al. 2015; Liu et al. 2016). Therefore, we next set out to experimentally test the hypothesis generated by our in silico analysis of the single-cell RNA-seq data that the two NECAB calcium-binding proteins are indeed characterizing CB_1_/CCK-positive interneurons at both the mRNA and protein levels.

To visualize *Necab1* and *Necab2* mRNA distribution, we used multichannel RNAscope fluorescent ISH, a highly specific and sensitive technique to detect even low copies of mRNA molecules in fixed tissues (Wang et al. 2012). To determine the localization of NECAB1 and NECAB2 proteins, we performed free-floating immunostaining by using antibodies raised against different epitopes of the two NECAB proteins. The specificity of the NECAB1 and NECAB2 antibodies have been validated in the dorsal spinal horn by using antigen adsorption tests and NECAB2-knockout mice (Zhang et al. 2014, 2018). However, to further validate the ISH and immunostaining experiments in the absence of NECAB1 and NECAB2 knockout mice in our laboratory, we combined the RNAscope protocol with immunostaining that allows the simultaneous detection of mRNA molecules and proteins in brain sections. We argued that it is highly unlikely that the two different sets of probes and experimental approaches would cause identical background patterns.

**Figure 2:**
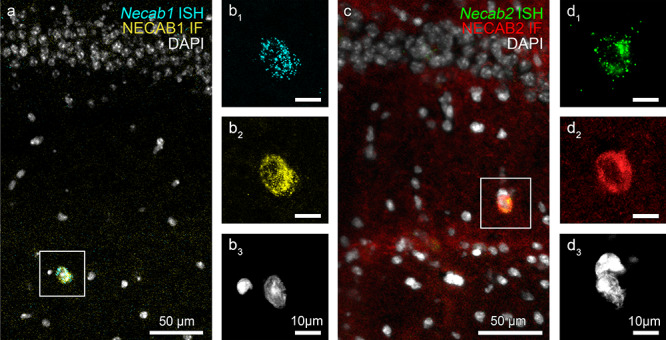
Colocalization of *Necab1/2* RNAscope ISH signals and NECAB1/2 immunofluorescence staining (IF) pattern in hippocampal interneurons. (*a*–*d*) Combined ISH and IF visualizes *Necab1/2* mRNA and NECAB1/2 protein distributions, respectively, and indicate the specificity of riboprobes and antibodies. Nuclei are stained with DAPI (white). (*a*) Low magnification confocal image shows a *Necab1* mRNA-expressing (cyan) and NECAB1 protein-containing interneuron (yellow) in the stratum radiatum of the CA1 subfield of the hippocampus. (*b*_1_–*b*_3_) The same interneuron from the boxed area in (*a*) is depicted at higher magnification. (*c*) *Necab2* mRNA (green) and NECAB2 protein (red) are also found to colocalize in a large stratum radiatum interneuron. (*d*_1_–*d*_3_) High magnification confocal image demonstrates high *Necab2*/NECAB2 levels within the same interneuron from the boxed area presented in (*c*).

Notably, RNAscope ISH and immunostaining both visualized scattered interneuron cell bodies in the hippocampus (Fig. 2). All NECAB1-immunopositive interneuron cell bodies did also contain *Necab1* mRNA signals in the stratum radiatum of the CA1 subfield (*n* = 61/61 cells from 3 mice). Similarly, every NECAB2-immunostained soma was also positive for *Necab2* expression (*n* = 82/82 cells from 3 mice). The abundance of GAD67, a primarily presynaptic synthesizing enzyme of GABA reached the detection threshold of immunofluorescence in the vast majority of NECAB1- and NECAB2-immunopositive cell bodies (55 out 69 cells and 31 out of 38 cells in case of NECAB1 and NECAB2, respectively, *n* = 3 mice) corroborating that these cells are GABAergic interneurons (Supplementary Fig. 4).

**Figure 3:**
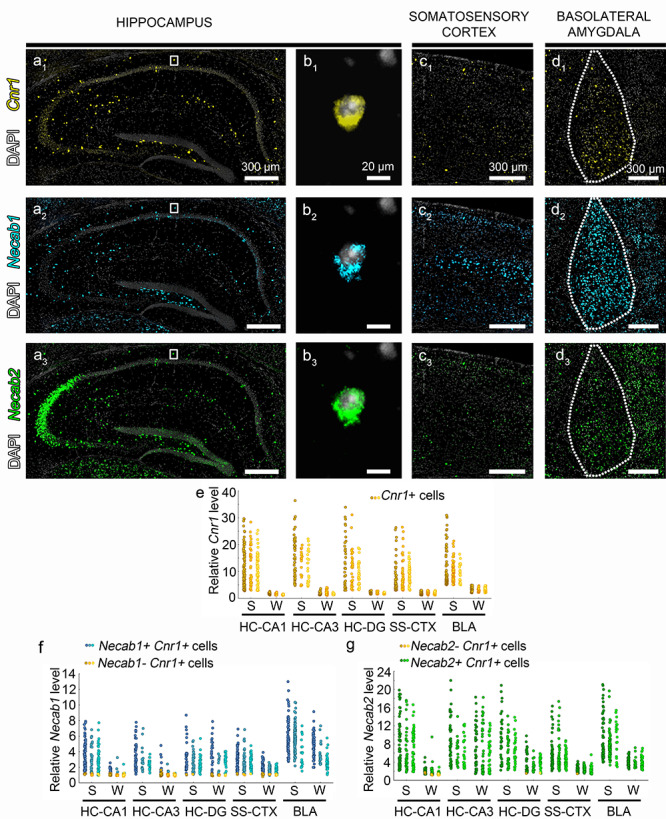
Prominent *Necab1 and Necab2* mRNA expression in high *Cnr1*-expressing hippocampal, cortical, and amygdalar interneurons. (*a*–*d*) Representative confocal microscopy images of triple fluorescent ISH shows the expression patterns of genes encoding the CB_1_ cannabinoid receptor (*Cnr1*, yellow), NECAB1 (*Necab1*, cyan), and NECAB2 (*Necab2*, green) in the mouse hippocampus (*a*_1_–*b*_3_), somatosensory cortex (*c*_1_–*c*_3_), and BLA complex (*d*_1_–*d*_3_). Cell nuclei are stained with DAPI (white) in all images. (*b*_1_–*b*_3_) High magnification of a triple-positive hippocampal interneuron located in the CA1 stratum oriens is shown from the boxed region in (*a*_1_–*a*_3_). Dashed line in (*d*_1_–*d*_3_) outlines the BLA complex. (*e*–*g*) Quantitative analysis of mRNA levels in 1337 individual cells segregates neurons into two major populations throughout the hippocampus (HC), somatosensory cortex (SS), and BLA complex. Cells exhibiting strong (S) Cnr1 mRNA expression are predominantly represent GABAergic interneurons, whereas neurons with weak (W) Cnr1 mRNA levels belong to principal cells. The three columns of data points represent individual cells from *n* = 3 mice. Note that both Necab1 and Necab2 are consistently highly expressed in strong Cnr1-positive cells in all three regions. Interestingly, weak Cnr1-positive cells that are putative mossy cells in the hilus of the DG also express moderate levels of both Necabs. Specific pyramidal cell populations express Necab1 in the somatosensory cortex and in the BLA complex, whereas Necab2 has high levels in CA2 and CA3a pyramidal neurons.

The potential crosstalk between the two riboprobes or antibodies could be excluded as well, because these tools also labeled specific, nonoverlapping subsets of excitatory cells. Besides the putative GABAergic interneurons, *Necab1* mRNA and NECAB1 protein were found in layer 2 and layer 5a pyramidal neurons in the neocortex, whereas *Necab2* mRNA and NECAB2 protein were observed in CA2 and CA3a/b pyramidal neurons in the dorsal hippocampus (Fig. 3).

CB_1_/CCK-positive interneurons belong to a GABAergic cell class that originate from the CGE and disperse via multiple rostro-caudal migratory streams to populate the isocortex, hippocampal formation, and BLA complex (Nery et al. 2002; Touzot et al. 2016). High CB_1_ level is a consistent feature of CB_1_/CCK-positive interneurons in all three telencephalic areas (Katona et al. 1999, 2001; Marsicano and Lutz 1999; Tsou et al. 1999; McDonald and Mascagni 2001; Bodor et al. 2005). On the other hand, CB_1_ is also present in glutamatergic excitatory cells albeit at an order of magnitude lower levels (Marsicano and Lutz 1999; Steindel et al. 2013). Analysis of the single-cell RNA-seq dataset verified this striking quantitative difference between the two major cell types (Supplementary Fig. 5).

To experimentally determine if *Necab1* and *Necab2* are expressed in CB_1_/CCK-positive interneurons, we applied 3-channel (*Cnr1*, *Necab1*, *Necab2*) RNAscope and used the quantitative nature of the RNAscope signals. Individual cells were categorized into “strong CB_1_-expressing” and “weak CB_1_-expressing” group based on the bimodal distribution of the *Cnr1* RNAscope signal intensity (for enrichment value cutoffs, see Materials and Methods). In the CA1 subfield of the hippocampus, we found that 92% and 100% of the “strong CB_1_-expressing” interneurons also express high levels of *Necab1* and *Necab2*, respectively (*n* = 194 cells from 3 mice; Fig. 3*a,b,e,f,g*). Similarly, all “strong CB_1_-expressing” interneurons in the CA3 region were also *Necab1-* and *Necab2*-positive (*n* = 93 cells from 3 mice; Fig. 3*a,b,e,f,g*). In the hilus of the dentate gyrus (DG), 97% of the “strong CB_1_-expressing” cells expressed *Necab1*, whereas all cells contained *Necab2* (*n* = 105 cells from 3 mice; Fig. 3*a,b,e,f,g*). “Weak CB_1_-expressing” pyramidal cells had few *Necab* signal in the CA1 (*n* = 141 cells from 3 mice; Fig. 3*a,e,f,g*). In contrast, *Necab2*, but not *Necab1* was found to be highly expressed in a selected subset of pyramidal neurons in the CA2/CA3 region (*n* = 123 cells from 3 mice; Fig. 3*a,e,f,g*). Interestingly, *Necab1* and *Necab2* were found in 80% and 97%, respectively, of “weak CB_1_-expressing” cells in the hilus (*n* = 74 cells from 3 mice; Fig. 3*a,e,f,g*) that may represent hilar mossy cells (Katona et al. 2006; Monory et al. 2006).

To investigate whether high levels of *Necab1* and *Necab2* are conserved molecular features of CB_1_/CCK-positive interneurons in light of their shared CGE origin, we carried out similar 3-channel RNAscope signal quantification in the somatosensory cortex and in the BLA (Fig. 3*c*–*g)*. We found that 97% and 100% of the “strong CB_1_-expressing” interneurons contain high levels of *Necab1* and *Necab2*, respectively, in the somatosensory cortex (*n* = 156 cells from 3 mice). We observed a similar situation in the BLA, where all “strong CB_1_-expressing” cells had high *Necab1* and *Necab2* levels (*n* = 189 cells from 3 mice). The majority of putative “weak CB_1_-expressing” principal cells in the somatosensory cortex contained moderate levels of *Necab1* and *Necab2* RNAscope signals (*n* = 143 cells from 3 mice). In contrast, *Necab1* had surprisingly high levels in presumed pyramidal neurons belonging to the “weak CB_1_-expressing” group (*n* = 119 cells from 3 mice), whereas *Necab2* expression level was substantially lower in the same sample of cells in the BLA complex.

We also addressed the issue whether high levels of *Necab1* and *Necab2* are restricted exclusively to CB_1_/CCK-positive interneurons. This analysis was carried out in the hippocampus, because intermingling of some indistinguishable high *Necab*-expressing pyramidal neurons and GABAergic interneurons would have increased false negative rate within layer 5 of the somatosensory cortex and in the BLA. We found that very few *Necab1*-and *Necab2*-positive interneurons lacked high levels of *Cnr1* RNAscope signal in both the CA1 (*n* = 3 and 25 out of 338 and 360 cells, respectively, from 3 mice) and CA3 subfields (*n* = 6 and 12 out of 222 and 228 cells, respectively, from 3 mice). Taken together, these RNAscope ISH experiments provide direct experimental evidence that the *Necab1* and *Necab2* genes encoding two EF-hand calcium-binding proteins are commonly expressed in CB_1_/CCK-positive interneurons in cortical microcircuits.

**Figure 4:**
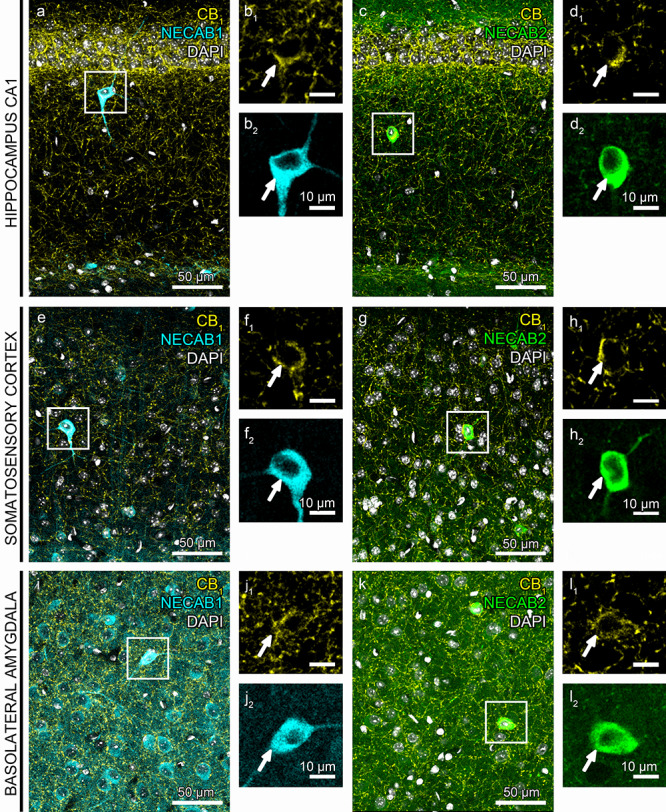
NECAB1 and NECAB2 calcium-binding proteins are distributed in CB_1_-receptor-expressing GABAergic interneurons. (*a*–*l*) Representative confocal microscopy images of double immunostaining of CB_1_ receptors (yellow) and either NECAB1 (cyan) or NECAB2 (green). Cell nuclei are stained with DAPI (white). Interneurons colocalizing CB_1_/NECAB1 or CB_1_/NECAB2 stand out already at low magnification in the CA1 subregion of the hippocampus CA1 region (*a*, *c*), in the somatosensory cortex (*e*, *g*) and in the BLA complex (*i*, *k*). (*b*_1_–_2_, *d*_1_–_2_, *f*_1_–_2_, *h*_1_–_2_, *j*_1_–_2_, *l*_1_–_2_) High magnification shows the double-immunostained cell bodies from the respective boxed areas present in *a*, *c*, *e*, *g*, *i*, *k*. Note that both CB_1_ receptor- and NECAB2-immunostaining visualize a dense meshwork of axons in all three regions, whereas NECAB1 is primarily located in somata and dendrites.

To determine whether NECAB1 and NECAB2 proteins are also present in CB_1_/CCK-positive interneurons, we performed combined immunostaining for CB_1_ receptors and either NECAB1 or NECAB2 and then measured colocalization ratios by dual-channel confocal microscopy (Fig. 4). NECAB1-immunostaining resulted in strong labeling of the somatodendritic compartment of multipolar cells in all examined brain regions (Fig. 4*a,b,e,f,i,j*), but putative principal cell bodies have also been noticed, especially in layer 5a of the somatosensory cortex and in the BLA (Fig. 4*e,i*). In case of NECAB2-immunostaining, the scattered distribution of putative interneuron cell bodies was complemented with an intense axonal arborization pattern throughout all three brain areas (Fig. 4*c,d,g,h,k,l*). CB_1_-immunostaining revealed the well-described dense meshwork of axons and large axon terminals, whereas interneuron cell bodies were also visualized by punctate, granular staining (Fig. 4*b,d,f,h,j,l*). The colocalization analysis showed that all CB_1_-immunopositive cell bodies are also immunostained for NECAB1 (*n* = 221 cells in the CA1 subfield; *n* = 237 cells in the CA3 subfield; *n* = 154 cells in the DG; *n* = 153 cells in the somatosensory cortex; and *n* = 151 cells in the BLA from 3 mice); and for NECAB2 (*n* = 97 cells in the CA1 subfield; *n* = 50 cells in the CA3 subfield; *n* = 96 cells in the DG; *n* = 132 cells in the somatosensory cortex; and *n* = 114 cells in the BLA from 3 mice).

In addition, we have also performed triple immunostaining for CB_1_, CCK, and either of the two NECAB calcium-binding proteins (Supplementary Fig. 6). Because CCK, as a neuropeptide is predominantly accumulated in the axon terminals, the somatic levels of CCK often remain below the detection threshold of immunolabeling. Nevertheless, qualitative observations showed that those hippocampal interneurons that had visible amount of CCK- and CB_1_ receptor-immunolabeling in their cell bodies had strong NECAB1- and NECAB2-immunostaining as well (Supplementary Fig. 6). Collectively, these anatomical experiments demonstrate that both NECAB1 and NECAB2 are ubiquitous calcium-binding proteins in CB_1_/CCK-positive interneurons throughout the cerebral cortex, the hippocampal formation, and the BLA complex.

Finally, we aimed to determine whether the subset of high *Necab*-expressing cells that lack *Cnr1* expression may belong to another interneuron type. Parvalbumin is the most well-established interneuron marker and it is present in several types such as basket cells, chandelier/axo-axonic cells, bistratified cells, and oriens–lacunosum moleculare (O-LM) interneurons in the CA1 region of the hippocampus (Klausberger and Somogyi 2008). Double immunostaining for NECAB1 and parvalbumin revealed that a subpopulation of NECAB1-positive interneurons also contains parvalbumin (*n* = 66 out of 330 cells from 3 mice). These cells consist of 38% of all parvalbumin-containing interneurons in the strata pyramidale and oriens (Supplementary Fig. 7*a_13_–a*) and may represent chandelier/axo-axonic interneurons (see Discussion for details). In contrast, parvalbumin level did not reach detection threshold in 213 out of 214 NECAB2-immunopositive interneurons (3 mice, Supplementary Fig. 7*b_13_–b*).

### Different Subcellular Distribution of NECAB1 and NECAB2 in CB_1_/CCK-Positive Interneurons

**Figure 5:**
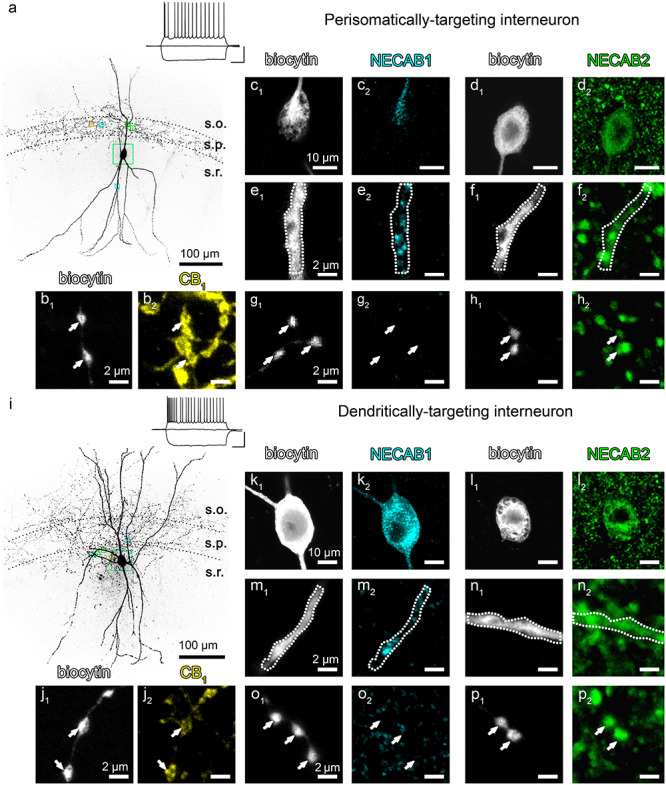
Subcellular distribution of NECAB1 and NECAB2 in identified perisomatically targeting and dendritically targeting CB_1_-receptor-expressing GABAergic interneurons. (*a*) MIP of a representative perisomatically targeting multipolar neuron in CA1 stratum radiatum (s.r.). The axonal arbor is restricted to stratum pyramidale (s.p.), whereas the dendrites are extended to stratum oriens (s.o.) and stratum radiatum. Boxed areas depict the different subcellular compartments that are shown at high magnification in *b*–*h* with matching colors. The neuron was filled with biocytin during patch-clamp recording. Insets show example voltage traces in response to depolarizing and hyperpolarizing current steps of −200, 0, and +150 pA from resting membrane potential recorded in whole-cell current-clamp configuration (scale: 40 mV, 200 ms). Note the regular-spiking, accommodating firing pattern, a characteristic feature of CB_1_/CCK-positive GABAergic interneurons. (*b*_1_–*b*_2_) Indeed, biocytin-filled axon terminals (*b*_1_) belonging to the same interneuron carry high density of CB_1_ receptors (yellow, *b*_2_). (*c*_1_–*d*_2_) The cell body of this interneuron was cut in half during resectioning (*c*_1_, *d*_1_) that makes the demonstration of the presence of both NECAB1 (cyan) and NECAB2 (green) within the soma possible (*c*_2_, *d*_2_). (*e*_1_–*f*_2_) Dendrites of the biocytin-filled cell also contain both NECAB1 (*e*_2_) and NECAB2 (*f*_2_). (*g*_1_–*h*_2_) In contrast, confocal imaging reveals intense NECAB2-immunostaining (*h*_2_) in axon terminals, whereas NECAB1-immunostaining remained under detection threshold in boutons of the same interneuron (*g*_2_). (*i*–*j*_2_) MIP of a confocal *z*-stack presents a regular-spiking dendritically targeting interneuron with an extensively distributed axonal arbor that contain CB_1_ receptors (*j*_2_). The subcellular distribution of the NECAB calcium-binding proteins within this cell mirrors their localization in the perisomatically targeting interneuron presented in (*a*). While the cell body and the dendrites contain both NECABs (*k*_1_–*n*_2_), only NECAB2-immunostaining is observed in the axon terminals (*p*_2_).

The previous findings pose the interesting question of why a single interneuron may need two phylogenetically closely related calcium-binding proteins? In light of the different kinetic properties of presynaptic and somatodendritic Ca^2+^-transients in regular-spiking CB_1_/CCK-positive interneurons in the CA1 area (Kisfali et al. 2013), we tested the hypothesis that the two NECAB proteins bear distinct subcellular compartmentalization and thereby regulate different Ca^2+^-mediated physiological processes. We first targeted multipolar, regular-spiking interneurons located in the stratum radiatum of the CA1 subfield of the hippocampus in acute brain slices by using whole-cell patch-clamp electrophysiology. After the identification of a regular-spiking interneuron, the cell was filled with biocytin to reconstruct its neuronal morphology. We studied two morphological types of CB_1_/CCK-positive interneurons that are known to exhibit some distinct molecular, anatomical, and electrophysiological properties (Lee et al. 2010, 2015; Dudok et al. 2015). The so-called perisomatically targeting interneurons (aka basket cells) innervate the cell body and proximal dendrites of postsynaptic pyramidal neurons (Fig. 5*a*), whereas the dendritically targeting (aka Schaffer collateral-associated) interneurons (Fig. 5*i*) form synapses on more distal pyramidal neuron dendrites (Vida et al. 1998; Cope et al. 2002). Every electrophysiologically recorded regular-spiking interneuron (*n* = 6 perisomatically targeting cells and *n* = 6 dendritically targeting cells) displayed high CB_1_ levels in its axon terminals (Fig. 5*b,j*).

**Figure 6:**
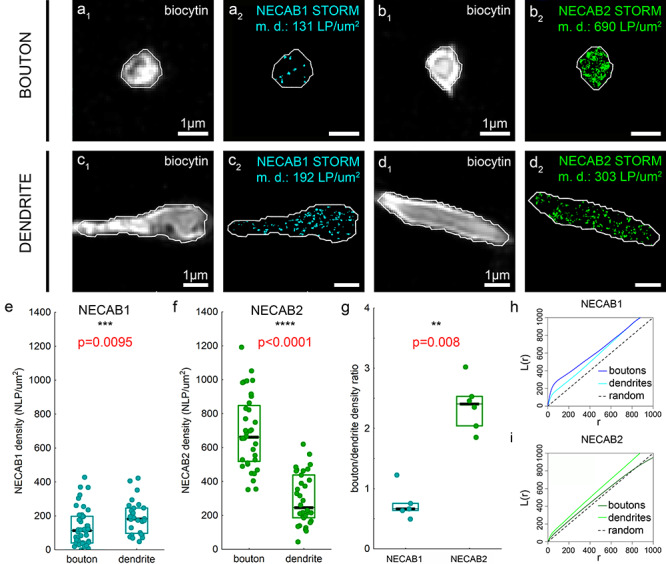
Correlated confocal and STORM super-resolution imaging reveals subcellular differences in the density of NECAB1 and NECAB2 calcium-binding proteins. (*a*–*d*) The distinct subcellular domains of identified perisomatically targeting interneurons were visualized by biocytin-labeling and confocal imaging (*a*_1_, *b*_1_, *c*_1_, *d*_1_). NECAB1- and NECAB2-immunostaining were imaged by STORM super-resolution microscopy within the very same subcellular profiles (*a*_2_, *b*_2_, *c*_2_, *d*_2_). To determine the nanoscale density of NECAB1 and NECAB2, the subcellular ROIs were delineated with the Active Contour algorithm (Marquez-Neila et al. 2014) that enabled filtering of those STORM coordinates that belong to the respective bouton or dendrite segment. (*e*) Based on the analysis of 43 axon terminals and 28 dendritic profiles, NECAB1 density (NLP per 1 μm^2^ area) is higher in the dendrites compared with boutons (*n* = 5 and 6 animals, respectively, Mann–Whitney *U*-test). (*f*) In contrast, NECAB2 density is substantially larger in axon terminals than in dendrites (*n* = 6 and 6 animals, 36 and 34 ROIs, respectively, Mann–Whitney *U*-test). (*g*) Accordingly, the ratio of the nanoscale density within the axonal and dendritic compartments is strikingly different between NECAB1 and NECAB2 with a predominant presynaptic accumulation of NECAB2 (*n* = 5 and 6 animals, Mann–Whitney *U*-test). Data are presented in *e*–*g* as median (black lines) and 25–75% interquartile range (cyan and green rectangles). (*h*) Ripley’s L-function of the nanoscale distribution of STORM localization points representing NECAB1 protein shows that NECAB1 has a nonuniform distribution and it is more clustered in axon terminals (blue) than in dendrites (cyan) (*n* = 6 and 5 animals, *n* = 51 and 40 ROIs, respectively). (*i*) Ripley’s L-function visualization demonstrates that NECAB2 nanoscale localization pattern is comparable with a uniform distribution in axon terminals (dark green), but it is more clustered in dendrites (light green) (*n* = 6 and 6 animals, *n* = 31 and 37 ROIs, respectively). Black dashed line in *h* and *i* shows the function for Poisson distribution (random).

In case of the NECAB1-immunostaining, we found that this calcium-binding protein is present throughout interneuron somata and dendrites (Fig. 5*c,e,k,m*). In contrast, NECAB1 levels remained under the detection threshold of confocal microscopy in the boutons of both perisomatically targeting and dendritically targeting interneurons (Fig. 5*g,o*). On the other hand, NECAB2-immunostaining was highly concentrated in the axon terminals (Fig. 5*h,p*), and was also found, albeit at lower levels, in the cell bodies and dendrites of both morphological types of CB_1_/CCK-positive interneurons (Fig. 5*d,f,l,n*).

Besides the qualitative difference in the subcellular localization of the two NECAB proteins, we also aimed to quantify the density of these calcium-binding proteins in both the somatodendritic and axonal compartments to gain better understanding of their nanoscale distribution. Conventional Ca^2+^-buffers of interneurons such as calretinin and parvalbumin have been shown to control Ca^2+^-signals in the nanodomain vicinity of voltage-gated Ca^2+^-channels that have important implications for presynaptic neurotransmitter release properties (Christel et al. 2012; Eggermann and Jonas 2012). Therefore, we next used super-resolution imaging as a powerful tool to measure the subcellular distribution of NECAB1 and NECAB2 in biocytin-filled perisomatically targeting cells. In contrast to confocal microscopy, the superior detection sensitivity of STORM imaging, as a type of single-molecule localization microscopy, was able to capture low levels of NECAB1 in interneuron axon terminals (Fig. 6*a*). However, NECAB1 density was still significantly higher in interneuron dendrites (Fig. 6*c,e*; Mann–Whitney *U*-test, *P* = 0.0095; 43 axonal and 28 dendritic ROIs from *n* = 6/5 cells and *n* = 6/5 mice, respectively). In striking contrast, NECAB2 protein was substantially more abundant in interneuron boutons (Fig. 6*b,f*) compared with interneuron dendrites (Fig. 6*d,f*; Mann–Whitney *U*-test, *P* < 0.0001; 36 axonal and 34 dendritic ROIs from *n* = 6/6 cells and *n* = 6/6 mice). Accordingly, quantification of the bouton/dendrite density ratio also revealed a considerable difference between the subcellular distribution of NECAB1 and NECAB2 (Fig. 6*g*; Mann–Whitney *U*-test, *P* = 0.008; *n* = 5/6 cells and *n* = 5/6 mice for NECAB1 and NECAB2, respectively) further supporting a hypothesized functional division of labor between these calcium-binding proteins.

Calcium-binding proteins involved in Ca^2+^-buffering can be mobile or immobilized and dynamic changes in the ratio of these two states underlie important physiological processes (Schwaller 2020). Therefore, we also investigated whether the NECAB calcium-binding proteins show a rather homogeneous distribution in axon terminals, cell bodies, and dendrites indicating a predominant function as mobile buffers; or alternatively, they are more concentrated within specific nanodomains in association with functional compartments such as presynaptic active zones, postsynaptic densities, or intracellular organelles like mitochondria. By exploiting the strength of STORM microscopy for nanoscale molecular imaging, we measured the clustering tendencies of the STORM localization points with Ripley’s L function (Fig. 6*h,i*). Apparent concentration hotspots distributed along the surface of the subcellular profiles that would indicate an exclusively plasma membrane-associated function were not observed (Fig. 6*a*–*d*). Nevertheless, Ripley’s L function detected deviations from spatial homogeneity in case of NECAB1 suggesting its clustered nanoscale distribution in both axon terminals and dendrites (Fig. 6*h*). Interestingly, while the nanoscale localization pattern of NECAB2 was only slightly different from random distribution in axon terminals, it tended to be more clustered in dendrites. Taken together, the subcellular compartment-specific nanoscale density and clustering differences between NECAB1 and NECAB2 indicate that these calcium-binding proteins fulfill multiple functionally different roles in the regulation of Ca^2+^-signaling dynamics in CB_1_/CCK-positive GABAergic interneurons.

## Discussion

The tight spatial and temporal regulation of Ca^2+^-signaling is pivotal in most neuronal physiological processes, whereas dysregulation of Ca^2+^-dynamics is often associated with brain disorders (Schwaller 2020). Accordingly, the immense molecular assortment of the “Ca^2+^-signaling toolkit” including the several hundred EF-hand Ca^2+^-binding proteins perfectly matches the cellular heterogeneity and the functional diversity of neurons throughout the brain (Girard et al. 2015). Our present findings obtained by in silico data mining in single-cell RNA-seq databases, ISH, immunostaining, and correlated confocal/super-resolution microscopy indicate the followings: (1) hippocampal CB_1_/CCK-positive GABAergic interneurons express high levels of N-terminal EF-hand calcium-binding protein 1 and 2; (2) the cellular expression pattern of NECAB1 and NECAB2 is a characteristic and conserved feature of developmentally and functionally related GABAergic cell populations in the somatosensory cortex and the BLA complex; (3) NECAB1 and NECAB2 exhibit different subcellular distribution in CB_1_/CCK-positive GABAergic interneurons. Together, these findings add to the growing body of evidence that separate molecular players were evolved to fine-tune neuronal calcium signaling at the cellular and subcellular levels and may subserve functional division of labor required for distinct Ca^2+^-dynamics in specific neuronal elements of cortical circuits.

### Interneuron-Type-Specific Anatomical Distribution of NECAB1 and NECAB2 Proteins

The cell-type-specific expression of representative EF-hand Ca^2+^-binding proteins such as parvalbumin, calbindin, and calretinin in cortical GABAergic interneuron types has been established more than three decades ago (Celio 1986; Baimbridge et al. 1992). The anatomical distribution of these Ca^2+^-buffers turned out to associate well with the postsynaptic target profile of interneurons either forming symmetrical synapses on different subcellular domains of pyramidal cells or specifying to innervate other interneurons (DeFelipe 1997; Kawaguchi and Kubota 1997; Freund and Buzsáki 1998). Early anatomical reports revealed that parvalbumin is predominantly restricted to basket and axo-axonic cells that target the perisomatic surface of principal cells in cortical and hippocampal microcircuits (Kosaka et al. 1987; Katsumaru et al. 1988; DeFelipe et al. 1989; Hendry et al. 1989). In addition, calbindin-containing interneurons were found to synapse primarily on dendritic shafts or project to the medial septum (Tóth and Freund 1992; Gulyás and Freund 1996; DeFelipe 1997), whereas a peculiar interneuron type that specifically innervates other interneurons was visualized by calretinin-immunostaining (Gulyás et al. 1996). Although later studies uncovered interneuron types that coexpress some of these Ca^2+^-binding proteins such as the parvalbumin- and calbindin-positive multipolar bursting cells in layer 2/3 of the cerebral cortex (Blatow et al. 2003), there is a general view that each GABAergic interneuron type expresses a characteristic and functionally relevant repertoire of Ca^2+^-buffers.

In light of the vast heterogeneity of cortical GABAergic cell types (Klausberger and Somogyi 2008; Tremblay et al. 2016; Pelkey et al. 2017), an important objective of our study was to ascertain if additional EF-hand Ca^2+^-binding proteins exist that control Ca^2+^-signaling in an interneuron-type-specific manner. Interestingly, prior anatomical studies have identified hippocampal and cortical interneuron types that innervate the cell bodies of pyramidal neurons and express the neuropeptide cholecystokinin, but lack parvalbumin (Greenwood et al. 1981; Harris et al. 1985; Nunzi et al. 1985; Freund et al. 1986; Kosaka et al. 1987). It is now well-established that these cells represent a highly heterogeneous group of GABAergic interneurons that provide complete coverage of different subcellular domains of principal cells by GABAergic inputs as described in the hippocampus and the neocortex (Freund et al. 1986; Klausberger et al. 2005; Lasztóczi et al. 2011), and extensively innervate the entire surface of pyramidal cells in the BLA complex (Rovira-Esteban et al. 2017). These cortical, hippocampal, and amygdalar interneurons share a common origin in the CGE (Nery et al. 2002). Together with CCK, their additional highly expressed and conserved molecular marker is the CB_1_ cannabinoid receptor (Katona et al. 1999, 2001; Marsicano and Lutz 1999; Bodor et al. 2005). A subpopulation of CB_1_/CCK-positive interneurons containing calbindin or occasionally calretinin were noted (Gulyás et al. 1991; Kubota and Kawaguchi 1997; Mascagni and McDonald 2003; Bodor et al. 2005; Rovira-Esteban et al. 2017) in complete agreement with the in silico expression data obtained in the present study. However, none of the three representative EF-hand Ca^2+^-binding proteins exhibit consistently high expression in CB_1_/CCK-positive interneurons.

The data mining approach showcases the usefulness of publicly available single-cell RNA-seq databases for hypothesis-driven anatomical studies and was instrumental to identify the two EF-hand Ca^2+^-binding proteins NECAB1 and NECAB2 as representative genes in CB_1_/CCK-positive interneurons. By using the most sensitive RNAscope and confocal imaging approaches, we verified an almost complete colocalization of *Necab*s and NECABs both at the mRNA and protein levels, respectively. The striking overlap indicates the uniform anatomical distribution of both NECABs in almost all CB_1_/CCK-positive interneurons. The lack of apparent colocalization in some cells found infrequently may result from methodical issues such as the limited detection threshold of immunostaining or the use of a single optical section for microscopic analysis. In addition, we provided direct evidence for the presence of NECABs in all investigated regular-spiking hippocampal CB_1_/CCK-positive interneurons at the level of identified perisomatically targeting and dendritically targeting interneurons by using confocal and super-resolution imaging. The anatomical distribution pattern of these interneurons is also in general agreement with previous ISH studies that showed scattered distribution of *Necab1*- and *Necab2*-expressing cells in different layers of the hippocampus (Sugita et al. 2002; Zimmermann et al. 2013). While these studies reported inconsistent findings regarding colocalization with calbindin, the presence of NECABs in the small subpopulation of calbindin-expressing CB_1_/CCK-positive interneurons described above could resolve this paradox (Gulyás et al. 1991). Notably, our in silico data also revealed a complete lack of parvalbumin, the occasional presence of calbindin and consistently high levels of both NECABs in every high CB_1_-expressing GABAergic interneuron.

Another important observation of the present study is that the high expression levels of both NECAB1 and NECAB2 in CB_1_/CCK-positive interneurons are conserved features in all three investigated telencephalic brain regions. The preserved anatomical distribution is also supported by a recent report describing the medial septal innervation of a few cells in the subiculum and parasubiculum that colocalize NECAB1 and CCK, but lack parvalbumin (Unal et al. 2015). It is imperative to emphasize that the CB_1_/CCK-positive population represents a significant, often underappreciated proportion of cortical GABAergic interneurons that matches the ratio of parvalbumin-positive interneurons among all GABAergic cells. Intriguingly, the percentage of CB_1_/CCK versus parvalbumin-positive interneurons varies in different microcircuits from 32% versus 16% in the dorsal CA1 to 8% versus 40% in the primary visual cortex, respectively (Whissell et al. 2015). Therefore, it was surprising that no typifying, highly expressed EF-hand Ca^2+^-binding proteins have been identified in such a major interneuron population to date. Our in silico and experimental findings that NECAB1 and NECAB2 are predominantly expressed by the CB_1_/CCK-positive interneurons fill this long-standing gap and contribute to the vast literature that different types of cortical, hippocampal, and amygdalar GABAergic interneurons express separate sets of Ca^2+^-binding proteins.

### Interneuron-Type-Specific Physiological Significance of NECAB1 and NECAB2 Proteins

Perhaps the best example for the cell-type-specific importance of Ca^2+^-buffering is described in the cerebellum. As shown by using global knockout and granule-cell-restricted rescue mouse models, the cytosolic Ca^2+^-buffering capacity of calretinin in cerebellar granule cells controls intrinsic neuronal excitability and firing pattern, thereby fine-tuning the frequency of cerebellar network oscillations and motor coordination behavior (Schiffmann et al. 1999; Gall et al. 2003; Cheron et al. 2004; Bearzatto et al. 2006). It is very interesting to consider why NECAB1 and NECAB2 were evolved as regulators of Ca^2+^-signaling in CB_1_/CCK interneurons. We observed a ubiquitous expression pattern in CB_1_/CCK interneurons throughout the cerebral cortex, the hippocampal formation, and the BLA complex suggesting that the physiological significance of NECAB1/2-mediated control of Ca^2+^-signaling should be considered in the context of the computational functions of these interneurons in circuit activity. However, the specific circuit and behavioral roles of CB_1_/CCK-positive interneurons have remained rather elusive. The incomplete and inaccurate cell-type-specific expression of genetic regulatory tools in CB_1_/CCK-positive interneurons renders in vivo experimental manipulation of their physiological and behavioral functions difficult (Rovira-Esteban et al. 2019). Importantly, coexpression of both NECAB Ca^2+^-binding proteins is restricted to the CB_1_/CCK-positive interneurons among cortical and hippocampal GABAergic cells indicating the potential usefulness of their genes for cell-type-specific labeling and interrogating of CB_1_/CCK-positive interneurons for future investigations. Intersectional genetic manipulation strategies will be required because we have also found that both NECABs are present in some selected, but nonoverlapping cortical and hippocampal pyramidal neuron types. The triple-recombinase-responsive approach (Plummer et al. 2015) including a GABAergic neuron-restricted promoter could be considered especially in the BLA, due to the widespread *Necab* expression in principal cells. In addition, an intersectional approach using the *Pvalb* and *Necab1* genes could potentially be exploited to target chandelier (axo-axonic) cells. Although we observed that most NECAB1-positive interneurons lack parvalbumin as reported earlier (Sugita et al. 2002; Zimmermann et al. 2013), we also found that NECAB1, but not NECAB2 is present in a smaller subset of parvalbumin-positive interneurons. Chandelier cells are distinguished from parvalbumin-positive basket cells based on the absence of *Satb1* transcription factor expression (Viney et al. 2013). Although further experimental evidence is required to establish whether the NECAB1/parvalbumin-immunopositive cells indeed represent chandelier cells, it is suggestive that the new Allen Institute single-cell RNA-seq dataset based on ~1.2 million cortical cells (Yao et al. 2020) contains a unique population of parvalbumin-positive interneurons that lack *Satb1*, and express *Necab1*, but not *Necab2*.

Although NECABs have been cloned almost 20 years ago (Bernier et al. 2001; Sugita et al. 2002), our knowledge about their cell physiological functions is still very limited. By illuminating the subcellular and cellular contexts in which NECAB-mediated Ca^2+^-buffering may have physiological significance, our present anatomical identification of the interneuron population and the subcellular domains that contain high levels of the NECAB Ca^2+^-binding proteins will also help to orient future studies that aim to uncover the cell physiological roles of NECABs by using loss-of-function approaches. In line with our observations in telencephalic brain regions, NECAB proteins also showed a cell-type-restricted distribution pattern that did not overlap with the other conventional calcium-binding proteins in the spinal cord and in the dorsal root ganglia (Zhang et al. 2014, 2016, 2018). The largely nonoverlapping distribution of individual Ca^2+^-binding proteins raise the interesting question of why CB_1_/CCK-positive interneurons utilize two related, but different Ca^2+^-binding proteins? One possibility could be the functional necessity of heterodimer formation between the two NECABs. On the other hand, our observations suggested a segregated distribution of NECAB1 and NECAB2 in different principal cell types. In addition, NECAB1 and NECAB2 are also present in different neuronal populations in the spinal cord implying their distinct physiological functions (Zhang et al. 2016). Moreover, the different subcellular distribution uncovered in the present study by STORM super-resolution imaging suggests a distinct contribution of NECAB1 and NECAB2 to somatodendritic and presynaptic Ca^2+^-dynamics in CB_1_/CCK-positive interneurons. As an important limitation of antibody-based immunostaining approaches, one must emphasize that the quantitative differences may partly arise from the altered immunogenicity of the two NECABs in the different subcellular environments in fixed tissue preparations. However, direct measurement of Ca^2+^-transients by two-photon laser microscopy in live CB_1_/CCK-positive interneurons provided compelling evidence for striking differences in dendritic and boutonal Ca^2+^-dynamics, such as monoexponential and biexponential decay of Ca^2+^-transients, respectively (Kisfali et al. 2013). Thus, it will be interesting to determine in future studies by using loss-of-function models such as viral-mediated targeted knockdown strategies of how NECAB1 and NECAB2 contribute to different Ca^2+^-kinetics in a subcellular compartment-specific manner.

The differential nanoscale distribution of NECAB proteins indicate that NECAB1 and NECAB2 are equipped with specific targeting and protein interaction motifs. Two previous studies have reported that NECAB2 specifically binds to A_2A_ adenosine receptors in the striatum (Canela et al. 2007), and interacts with mGlu_5_ glutamate receptor in hippocampal pyramidal cells (Canela et al. 2009). On the other hand, our STORM microscopy findings did not indicate a specific plasma membrane accumulation or synaptic clustering of neither Ca^2+^-binding proteins in dendrites. Instead, our nanoscale distribution analysis in biocytin-filled interneurons imply a predominantly intracellular localization, which is more in line with the electron micrographs depicting immunogold particles representing NECAB2 distribution (Canela et al. 2007, 2009) leaving the question open about the specific cell physiological functions of NECABs in interneuron dendrites. Considering the dense accumulation of NECAB2 that we observed in interneuron axon terminals, an interesting future direction of research may investigate how this Ca^2+^-binding protein may associate with presynaptic calcium-sensing proteins and how NECAB2-mediated Ca^2+^-buffering regulates neurotransmitter release. Both parvalbumin and calretinin are known to control Ca^2+^-transients in the nanoscale domain surrounding voltage-gated Ca^2+^-channels (Christel et al. 2012; Eggermann and Jonas 2012), even via the direct interaction with Ca^2+^-channel subunits (Christel et al. 2012). Binding of NECAB1 to the presynaptic calcium sensor synaptotagmin 1 has been reported, although its significance under more physiological conditions remains to be established (Sugita et al. 2002). On the other hand, our in silico correlation analysis identified *Syt6* encoding synaptotagmin 6, another calcium-sensing synaptic vesicle protein that exhibits strong positive correlation with *Necab2*, *Cnr1*, and *Cck* expression levels. Interestingly, two Patch-Seq-based single-cell RNA-seq studies have also measured strong expression of *Syt6* in CB_1_/CCK-positive interneurons (Földy et al. 2016; Fuzik et al. 2016). It is also important to note that parvalbumin-containing perisomatic axon terminals carry synaptotagmin 2 (Sommeijer and Levelt 2012), and exploit different voltage-gated Ca^2+^-channels than CB_1_/CCK-positive interneurons (Poncer et al. 1997; Wilson et al. 2001; Szabó, Lenkey, et al. 2014a). These observations together suggest that a specific set of Ca^2+^-channels, Ca^2+^-sensors and Ca^2+^-buffers controls Ca^2+^-dependent vesicle release and underlies the distinctive neurotransmitter release properties of CB_1_/CCK-positive interneurons (Hefft and Jonas 2005; Daw et al. 2009; Ali and Todorova 2010).

### Interneuron-Type-Specific Pathophysiological Implications of NECAB1 and NECAB2 Proteins

Presynaptic CB_1_ receptor-mediated control of the unusually long-lasting inhibitory inputs of principal cells distinguishes CB_1_/CCK-positive interneurons from parvalbumin-containing basket cells and contributes to their circuit-specific computational functions in neuronal ensemble formation (Freund and Katona 2007; Armstrong and Soltesz 2012; Bartos and Elgueta 2012). Novel experience alters endocannabinoid signaling and facilitates GABAergic inhibition in an activity-dependent manner in selected populations of principal cells that encode corresponding information traces (Hartzell et al. 2018; Sun et al. 2020). By setting the timing of activity of sparse neuronal ensembles, CB_1_/CCK-positive interneurons play an important role in establishing the proper balance between the discrimination and the generalization of memory traces (Sun et al. 2020). Intriguingly, impaired weighing between discrimination and generalization in perception and in other cognitively more complex processes such as social behavior culminates in over-selectivity and hypersociability that are core phenotypic features in autism spectrum disorders (Mottron et al. 2006; Brown and Bebko 2012; Church et al. 2015). Therefore, it is very important to highlight that de novo genetic lesions of NECAB2 are associated with idiopathic autism spectrum disorder (Sakai et al. 2011), whereas missense single nucleotide variants of NECAB1 are linked with developmental language disorders, a typical comorbidity in autism (Kornilov et al. 2016). Intriguingly, we also found in our in silico transcriptomic analysis of CB_1_/CCK-positive interneurons that *Necab*s expression levels show strong positive correlations with other autism-associated genes. For example, *Cadps2* (Calcium-Dependent Secretion Activator 2), is a gene whose protein product controls neurotransmitter release and its genetic variants and splicing errors are highly implicated in autism spectrum disorders (Cisternas et al. 2003; Sadakata et al. 2007; Bonora et al. 2014). Moreover, *Cxcl14* (C-X-C motif chemokine 14), a classifying gene for the largest population of CB_1_/CCK interneurons in both the hippocampus and isocortex (Harris et al. 2018; Yao et al. 2020) that encodes a chemokine regulating GABA release (Banisadr et al. 2011) is also among those genes whose expression is most affected in the dorsolateral prefrontal cortex of children with autism (Stoner et al. 2014).

In conclusion, our in silico and experimental anatomical findings unraveling NECAB1 and NECAB2 as prevalent calcium-binding proteins in CB_1_/CCK-positive interneurons together with the above genetic data and behavioral observations highlight the importance of Ca^2+^-signaling regulation in CB_1_/CCK-positive interneurons. We propose that a better understanding of the physiological functions of the two NECAB proteins and CB_1_/CCK-positive interneurons in general will also provide important insights into the aberrant circuit mechanism in autism spectrum disorders.

## Notes

The authors are grateful to Dr E. Horváth, B. Pintér, E. Tischler for laboratory support and to Dr S. Prokop and M. Zöldi for their comments on the manuscript. The help of Drs L. Barna, C. Pongor, P. Vági, the Nikon Microscopy Center at the Institute of Experimental Medicine, Nikon Europe B.V., Nikon Austria GmbH and Auro-Science Consulting is acknowledged for kindly providing microscopy support. The authors are indebted to Dr Masahiko Watanabe, to the CURE Gastroenteric Biology Center, to K. Lengyel and Dr B. Hangya; to Drs N. Hájos, G. Nyíri, and P. Papp for providing antibodies against the CB_1_ cannabinoid receptor, CCK, PV, and GAD67 respectively. *Conflict of Interest*: The authors declare no conflict of interest.

## Funding

New National Excellence Program of the Ministry of Human Capacities (ÚNKP-18-3); National Brain Research Program (2017-1.2.1-NKP-2017-00002); National Research, Development and Innovation Office, Hungary (Frontier Program 129961, KH124972); National Institutes of Health (R01NS099457, R01DA044925). I.K. also holds the Naus Family Chair in Addiction Sciences in the Department of Psychological and Brain Sciences at Indiana University Bloomington.

## Supplementary Material

Miczan_et_al_2020_Cerebral_Cortex_Supplementary_20201008_bhaa326
