# Supplementary material for: NECAB1 and NECAB2 are Prevalent Calcium-Binding Proteins of CB1/CCK-Positive GABAergic Interneurons
Source: Cereb Cortex. 2020 Nov 24;31(3):1786–806. doi: 10.1093/cercor/bhaa326 (PMC7869086; doi:10.1093/cercor/bhaa326)
Supplement: Miczan_et_al_2020_Cerebral_Cortex_Supplementary_20201008_bhaa326 [file miczan_et_al_2020_cerebral_cortex_supplementary_20201008_bhaa326.docx]

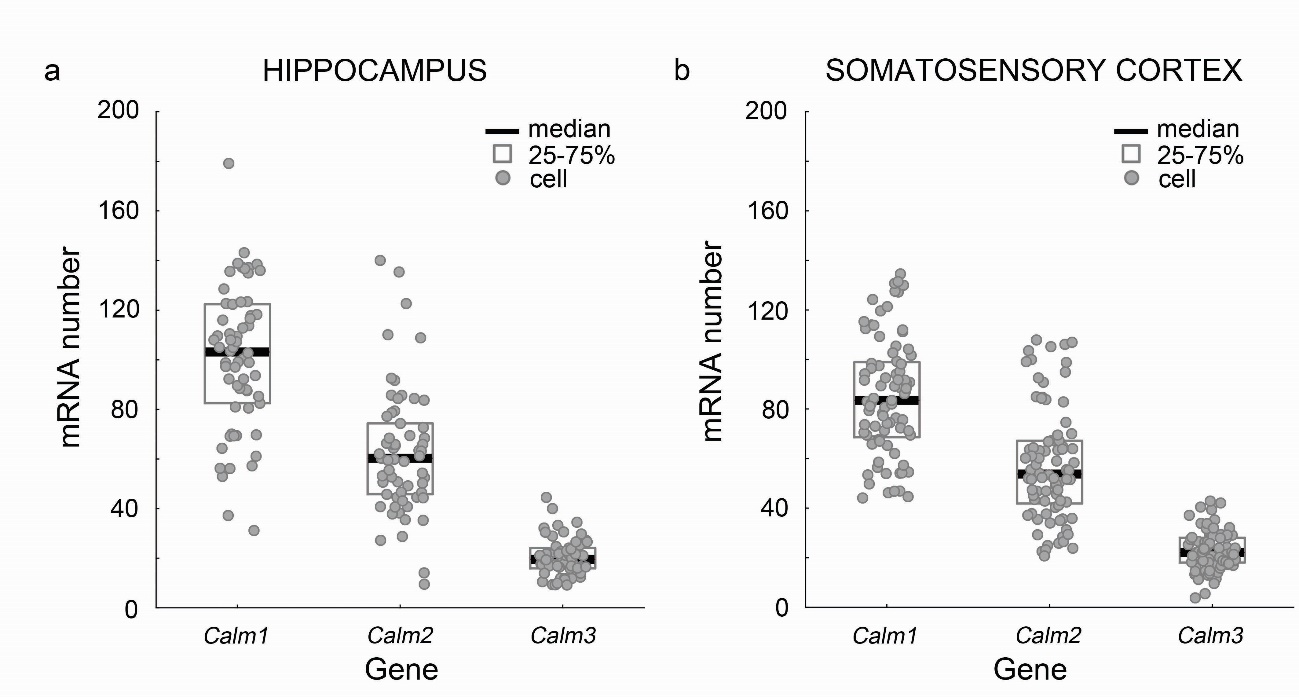


## Supplementary Figure 1. In silico analysis of the expression of calmodulin genes in CB_1_-receptor-expressing GABAergic interneurons.

## (a,b) The three calmodulin calcium-binding proteins are ubiquitous and abundant in all eukaryotic cells. In silico analysis of their mRNA expression levels in CB_1_ receptor-expressing interneurons serves as positive control. It verifies that the mRNA data obtained from the hippocampal (a) and cortical (b) single-cell RNAseq database (n = 61 and 84 cells, respectively, Zeisel et al. 2015) are amenable for gene expression analysis in individual GABAergic interneurons.


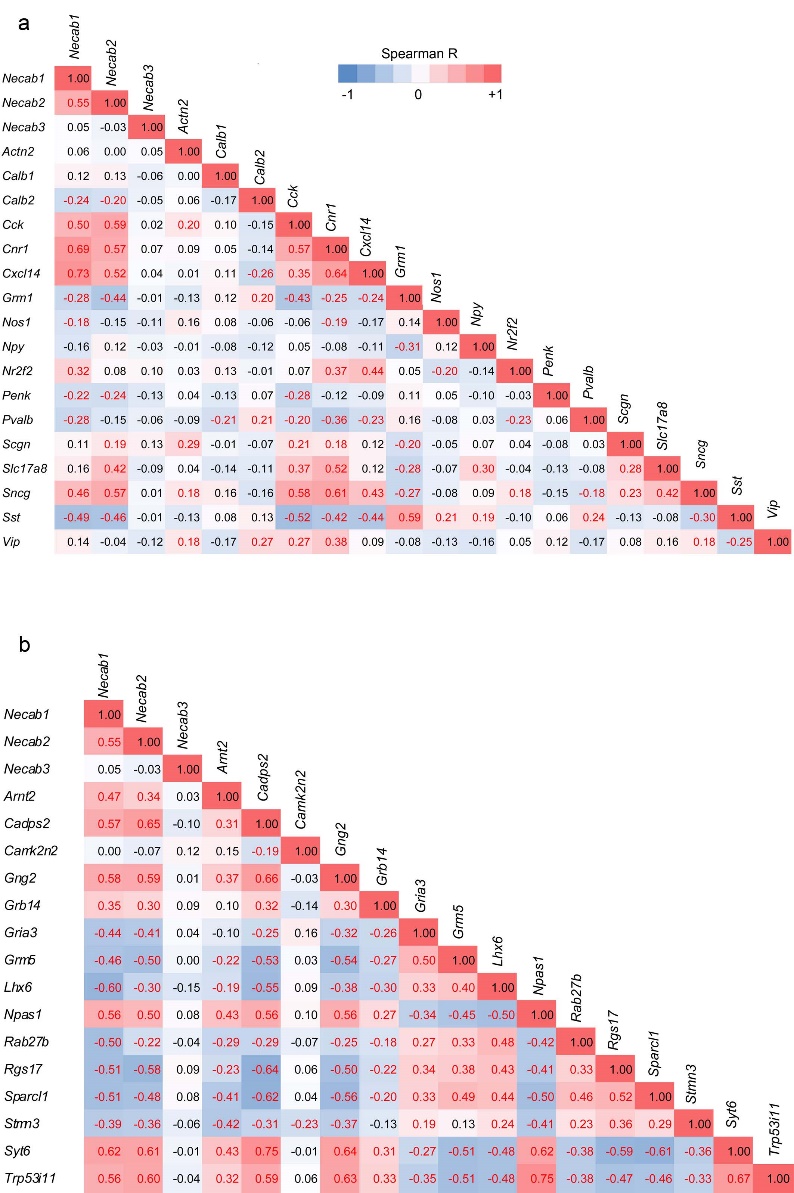


## Supplementary Figure 2. Correlation of Necab1 and Necab2 gene expression with distinct interneuron markers and related genes of interest.

## (a) Spearman’s rank correlation coefficient reflects the coexpression relationship (red) of Necab1 and Necab2 with well-known marker genes of CB_1_/CCK-positive GABAergic interneurons. Note the high positive correlation coefficient value with the Cnr1 (CB_1_) and Cck (CCK) genes. Negative correlation coefficient values (blue) with established marker genes of other interneuron types such as Sst (somatostatin) and Pvalb (parvalbumin) indicate the lack of co-expression in a sample of hippocampal interneurons (n = 126 cells from Zeisel et al. 2015). (b) Spearman’s rank correlation of gene expression of Necabs with other genes of interest identify genes that exhibit prominent positive and negative correlations with Necabs and are implied in interneuron-type-specific synapse function (see Discussion for details about Syt6 and Cadps2). Correlation coefficient values that are significant at p < 0.05 are highlighted with red text.


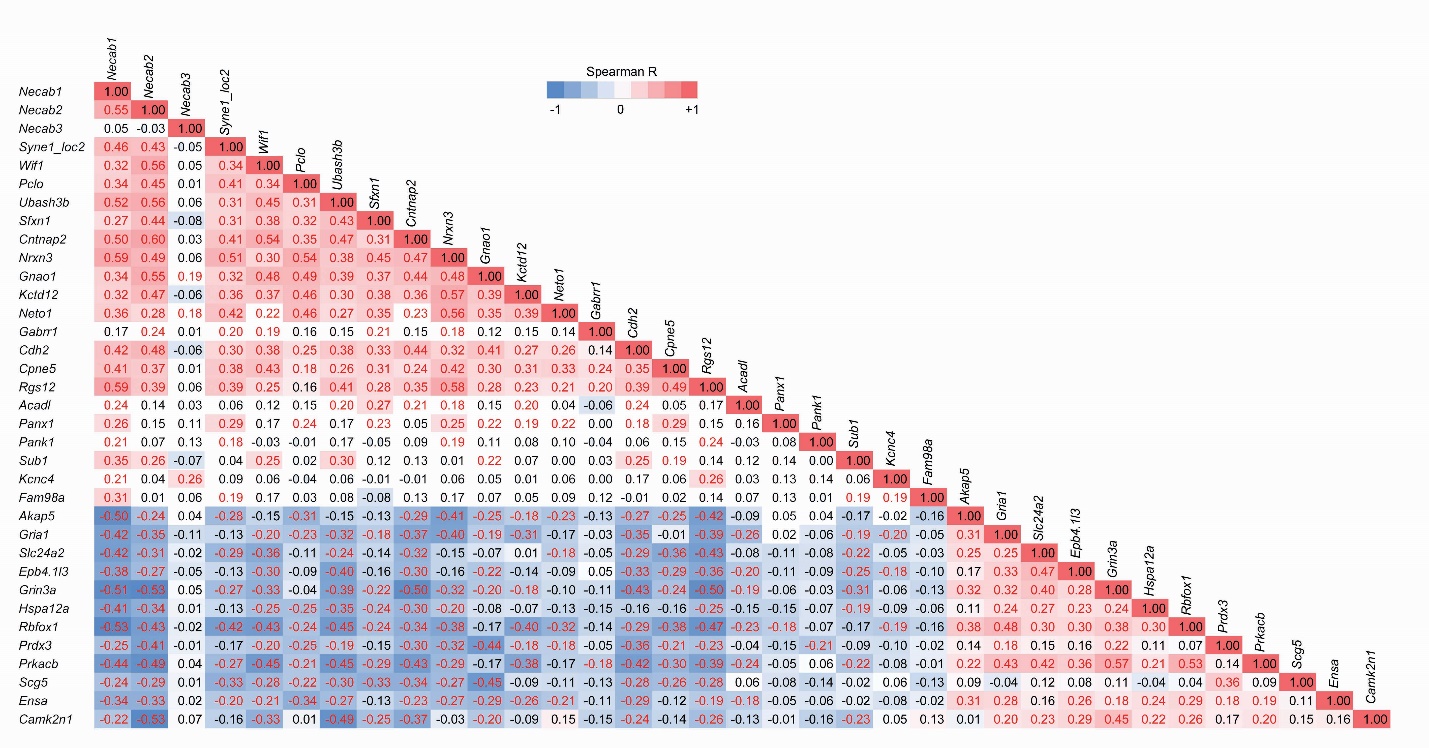


## Supplementary Figure 3. Genes showing high positive or negative correlation coefficient values with Necab1 and Necab2 in hippocampal interneurons.

## A selected set of genes are presented that show substantially high positive or negative Spearman’s rank correlation coefficient values with the mRNA levels of Necabs in hippocampal interneurons (n = 126 from Zeisel et al. 2015). Several of these genes have already been shown to be functionally important in CB_1_/CCK-positive GABAergic interneurons (such as Neto1, Pelkey et al. 2017), but the physiological role of most of these genes in hippocampal interneurons remains unknown. Values that are significant at p < 0.05 are highlighted with red text.


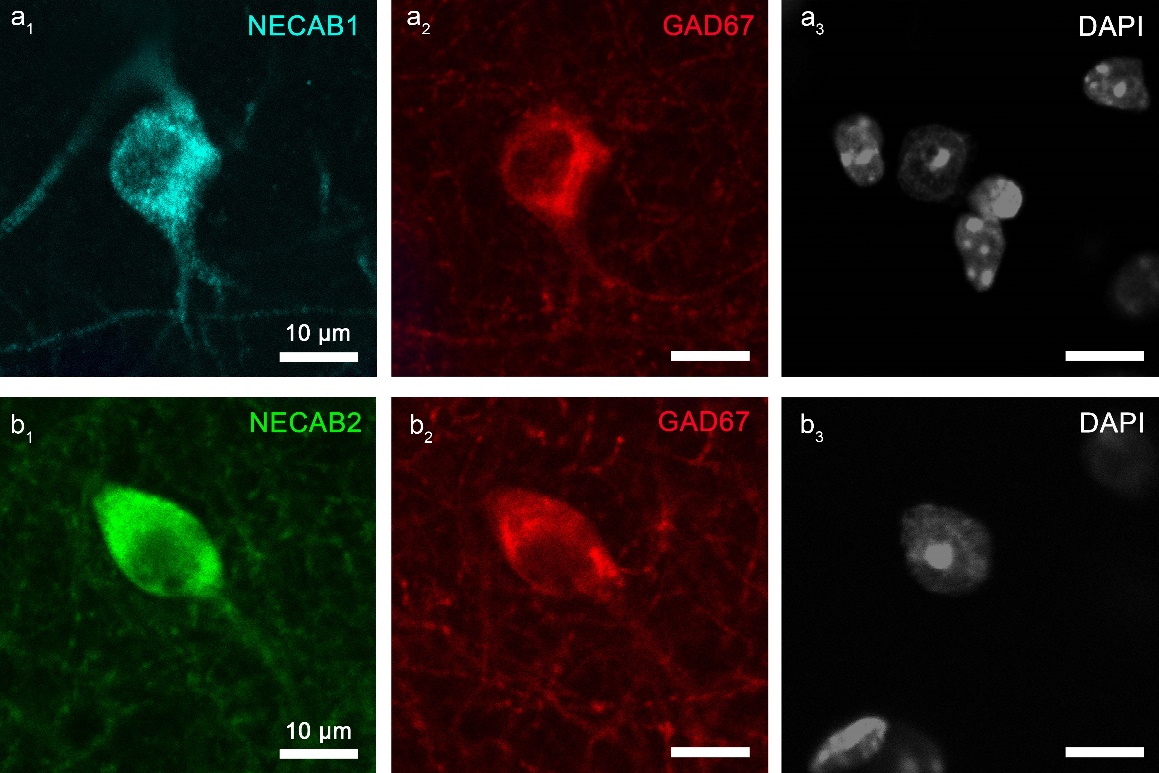


## Supplementary Figure 4. NECAB1 and NECAB2 colocalizes with GAD67 in hippocampal interneurons.

(a_1_-b_3_) Representative confocal images of double immunostaining of either NECAB1 (cyan) or NECAB2 (green) and GAD67 (red) obtained in CA1 stratum radiatum. Cell nuclei are stained with DAPI (white).


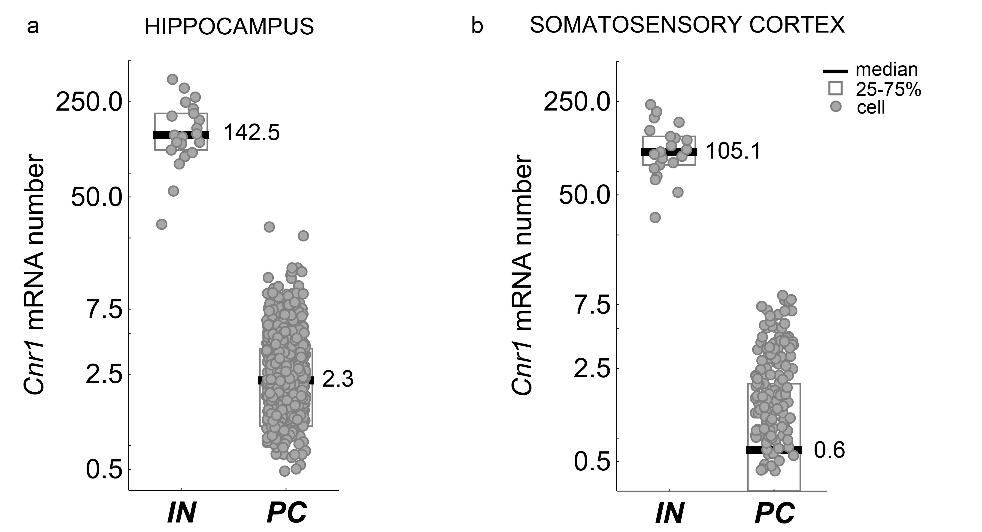


## Supplementary Figure 5. Prominent difference in Cnr1 mRNA levels between CB_1_/CCK-positive interneurons and pyramidal cells.

## (a,b) In silico analysis of Cnr1 mRNA expression profiles was performed on data derived from the single-cell RNAseq database of Zeisel et al. 2015. The samples were obtained from the mouse hippocampus (a) and somatosensory cortex (b). Cells in the database were selected based on their level 2 annotations into cell types (see Material and Methods). Number of interneurons in the CA1 subfield of the hippocampus: n = 21; in the somatosensory cortex: n = 21; number of principal cells in the CA1 subfield of the hippocampus: n = 827; in the somatosensory cortex: n = 285. Number of pyramidal cells with no detectable Cnr1 mRNA are n = 155 in the hippocampus and n = 133 in the somatosensory cortex. Data points for these cells are not shown but were considered in descriptive statistics. The y axes use logarithmic scale.


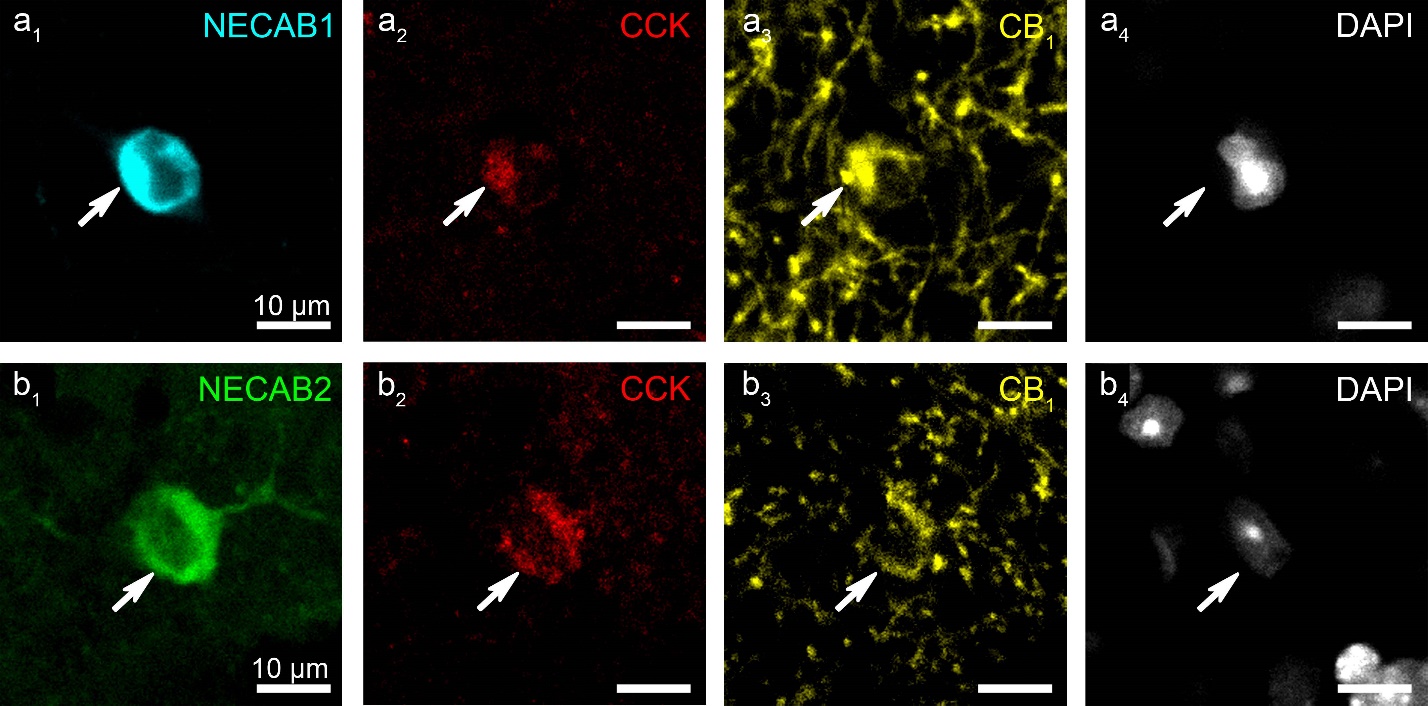


**Supplementary Figure 6. NECAB1 and NECAB2 calcium-binding proteins are present in CB_1_/CCK-positive GABAergic interneurons in the hippocampus.**

(a_1_-b_4_) Representative confocal microscopy images of triple immunostaining of CB_1_ receptors (yellow) and CCK (red) and either NECAB1 (cyan) or NECAB2 (green). Cell nuclei are stained with DAPI (white). The majority of CB_1_ and CCK proteins are transported to the axon terminals. However, some interneuron cell bodies have high enough CB_1_ and CCK levels and are visible by using immunostaining and confocal microscopy. Importantly, all of these cell bodies were also immunopositive for NECAB1 (a_1_-a_4_; n = 32 cells) and NECAB2 (b_1_-b_4_; n = 30 cells).


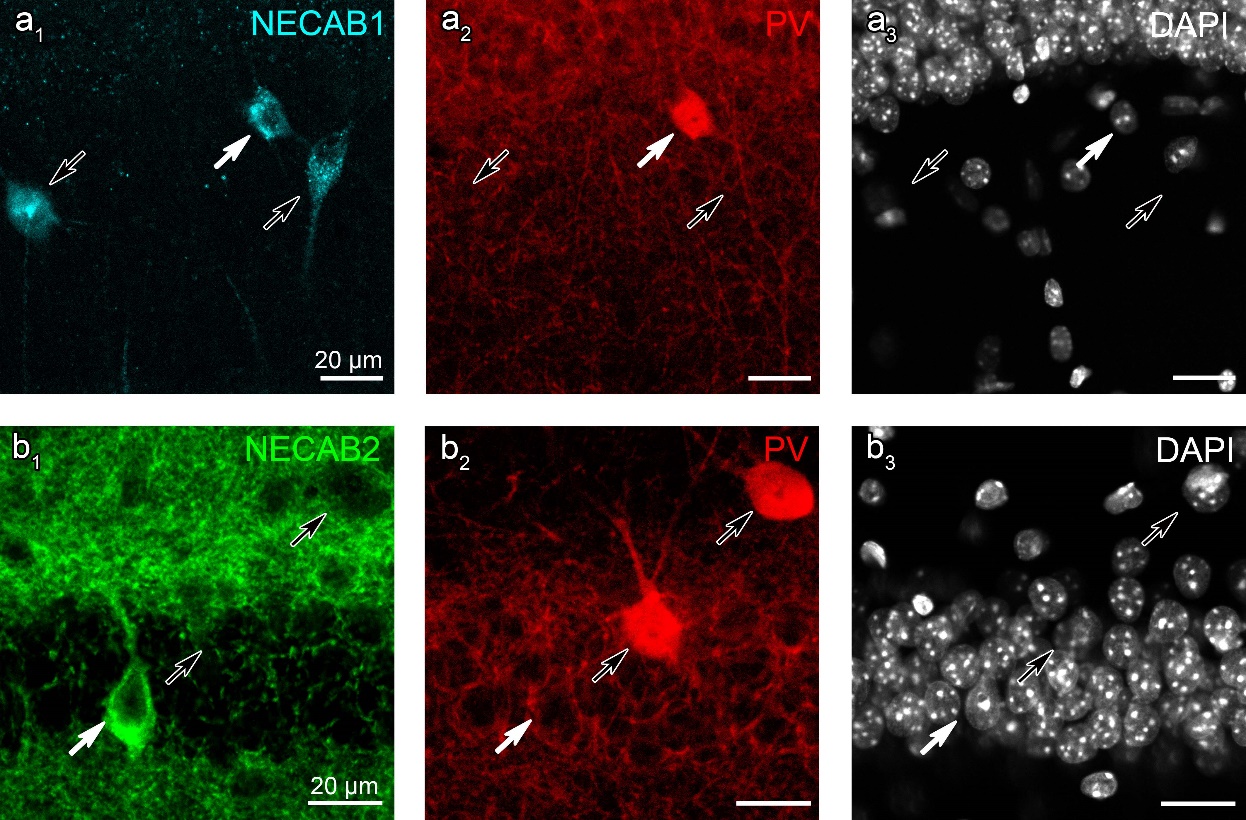


**Supplementary Figure 7. Parvalbumin colocalizes with NECAB1 in a subset of interneurons but it is absent in NECAB2-immunopositive cells.**

(a_1_-a_3_) Confocal images show double immunostaining of NECAB1 (cyan) and parvalbumin (PV, red). White arrow points to a PV-containing interneuron that contains NECAB1, whereas black arrows label PV-immunonegative, but NECAB1-immunopositive interneurons. (b_1_-b_3_) In the CA1 stratum pyramidale, NECAB2 (white arrow) and PV (black arrows) are located in different interneuron populations. Cell nuclei in a_3_ and b_3_ are stained with DAPI (white).
